# Amelioration of cyclophosphamide-induced DNA damage, oxidative stress, and hepato- and neurotoxicity by *Piper longum* extract in rats: The role of γH2AX and 8-OHdG

**DOI:** 10.3389/fphar.2023.1147823

**Published:** 2023-03-10

**Authors:** Vaishali Yadav, Anuja Krishnan, Sultan Zahiruddin, Sayeed Ahmad, Divya Vohora

**Affiliations:** ^1^ Neurobehavioral Pharmacology Laboratory, Department of Pharmacology, School of Pharmaceutical Education and Research, Jamia Hamdard University, New Delhi, India; ^2^ Department of Molecular Medicine, School of Interdisciplinary Science and Technology, Jamia Hamdard University, New Delhi, India; ^3^ Bioactive Natural Product Laboratory, Department of Pharmacognosy and Phytochemistry, School of Pharmaceutical Education and Research, Jamia Hamdard University, New Delhi, India

**Keywords:** *Piper longum* extract, cyclophosphamide, genomic instability, genoprotection, γH2AX, 8-OHdG

## Abstract

**Background:** The identification of genoprotectants is a promising strategy for improving human health. *Piper longum* has drawn scientific attention because of its diverse biological effects and traditional utilization. The current investigation aims to evaluate the genome-stabilizing potential of *Piper longum* against cyclophosphamide-associated genotoxicity.

**Methods:** We adopted a funnel screening with a three-tier evaluation approach, where *Piper longum* was investigated in an acellular medium, peripheral blood lymphocytes, and a rodent model. The genoprotective action of the *Piper longum* extract was initially performed with plasmid pBluescript SK(-) DNA. Furthermore, the extract and various fractions were screened against cyclophosphamide-induced genotoxicity using a cytokinesis-block micronucleus assay and a chromosomal aberration assay in human peripheral blood lymphocytes. The genome-stabilizing action of the extract and potent (hexane) fraction was further confirmed *in vivo* in Wistar albino rats by evaluating them using mammalian erythrocyte micronucleus tests, DNA fragmentation, oxidative stress markers, 8-hydroxy-2-deoxyguanosine (8-OHdG), γH2AX, and histopathological lesions in the liver and hippocampus. Additionally, acute and sub-acute toxicity studies were conducted following the Organization for Economic Co-operation and Development (OECD) guidelines for rats. Furthermore, the extract was quantified and characterized by high-performance thin-layer chromatography (HPTLC), ultra-high performance liquid chromatography–mass spectroscopy (UPLC-MS), and gas chromatography–mass spectrometry (GC-MS).

**Results:** The *Piper longum* ethanol extract was shown to protect plasmid pBluescript SK(-) DNA against H_2_O_2_-induced strand breaks. In human lymphocytes, the extract and hexane fraction showed a reduction in micronucleus formation (*p* < 0.001) and chromosomal aberrations (*p* < 0.01) against cyclophosphamide. Furthermore, the extract and fraction treatment, when administered at 200 mg/kg for 28 days in Wistar rats, restored cyclophosphamide-induced genomic instability by reducing micronucleus formation and DNA fragmentation; restoring redox homeostasis; decreasing 8-OHdG, a hallmark of oxidative DNA damage; reducing γH2AX, a DNA double-strand break (DSB) marker; and preserving the liver and hippocampus against histopathological lesions. The extract and fraction revealed no signs of systemic toxicity at the used doses. Piperine and piperlongumine are the major alkaloids quantified along with the presence of flavonoids in the ethanol extract and the presence of fatty acids and terpenoids in the hexane fraction of *Piper longum*.

**Conclusion:** Our investigation confirms the genoprotective action of *Piper longum* by reducing cyclophosphamide-associated cytogenotoxicity, oxidative stress, hepato- and neurotoxicity, oxidative DNA damage, and DNA double-strand breaks. The outcomes are critical for mitigating the genotoxic effects of chemotherapy recipients, requiring further attention.

## 1 Introduction

Genome instability refers to an elevated rate of DNA damage and associated mutations, arising from defects in DNA damage response, strand breaks in DNA, DNA replication, cell cycle control, or chromosome segregation (Kovalchuk, 2016). Several pathological conditions exhibit genome instability, such as aging, carcinogenesis, immune deficiencies, infertility, cardiovascular diseases, metabolic syndromes, and neuromuscular and neurodegenerative diseases (Georgoulis et al., 2017). Furthermore, human exposure to numerous genotoxicants has escalated considerably in recent years. Moreover, drugs such as cyclophosphamide are an important part of cancer treatment; their overexposure to patients undergoing chemotherapy causes high levels of DNA damage and generates secondary pathological events (oxidative stress, inflammatory response, and multiple-organ toxicity), which are closely associated with genomic instability and secondary tumor generation, thereby reducing the quality of life in cancer patients (Swift and Golsteyn, 2014). Several reports have further indicated that medical personnel chronically exposed to oncological medicines are at a greater threat of genome damage, and higher cytogenotoxic biomarkers are observed in clinical specimens (Vaghef et al., 1997; Rekhadevi et al., 2007; Moretti et al., 2011). As a result, a huge demographic is consistently exposed to genotoxins and its consequences. Thus, screening for genoprotective/genome-stabilizing agents will aid in reducing the adversity of diseases where the underlying pathology is associated with genomic instability, reducing the genotoxic impact/severe side effects for chemotherapy-receiving patients, defending health workers against genotoxic exposure of chemotherapeutics, and providing protection against environmental genomic damage.

*Piper longum L.*, a significant medicinal plant, is majorly utilized by indigenous medicinal systems of Asia and Pacific islands, particularly in India. The major secondary metabolites of *Piper longum*, piperine and piperlongumine, belong to the class of alkaloids. Apart from these, essential oils, bitter principle, steroids, coumarins, flavonoids, terpenoids, fatty acids, and sterols are also reported (Yadav et al., 2020). The ancient verse and Ayurvedic Pharmacopeia about *Piper longum* fruits suggest its action of improving the immune system and eliminating toxins from the body. Another application of *Piper longum* is in “Rasayana therapy,” a crucial Ayurvedic treatment dealing with rejuvenation, immunity boosting, and chemo-prevention (Praveenkumar et al., 1994; Govindarajan et al., 2005). The property of herbs to remove or neutralize the endotoxin might help to stabilize the genomic material, as endotoxins eventually cause the formation of harmful compounds within the biological system. Moreover, the scientific community has validated the efficacy of *Piper longum* as anti-cancer, immunomodulatory (Sunila and Kuttan, 2004), combating oxidative stress (Wakade et al., 2008), adaptogenic (Yadav et al., 2016), inhibitor of the cytochrome P450 enzyme (Harwansh et al., 2014), and radioprotective (Sunila and Kuttan, 2005). Reports suggest that the consequences of these biological actions along with the phytochemical composition of herbs might positively impact genome integrity (Luca et al., 2016; Izquierdo-Vega et al., 2017). Furthermore, small molecules, especially alkaloids, bind to DNA, either in grooves or intercalate into bases. The ability of compounds to interact with DNA is often associated with a DNA-protecting effect (Sjakste et al., 2020). Piperine (Haris et al., 2015), piperlongumine (Yadav et al., 2022), and other phytochemicals in the extract (Zahin et al., 2018) are known to interact with DNA grooves in molecular docking simulations and biophysical studies and are, therefore, implicated in genome protection.

Thus, the amalgamation of traditional and scientific evidence for *Piper longum* made it a suitable candidate to be screened as a genoprotectant. The aim of study, therefore, was to decrypt the potential of the *Piper longum* extract in models of genomic instability. Cyclophosphamide was used as an inducer of genomic instability, an alkylating agent commonly used in neoplastic diseases (Ogino and Tadi, 2020). We adopted a funnel screening process with a three-tier evaluation approach, where the behavior of the *Piper longum* extract was investigated in a cell-free medium, mammalian cells, and a genomic instability rodent model, using DNA topology, cytogenetic alterations, and biochemical markers of genotoxicity.

## 2 Results

### 2.1 Protective effects of the *Piper longum* ethanol extract on plasmid pBluescript SK(-) DNA against H_2_O_2_-induced strand breaks

**FIGURE 1:**
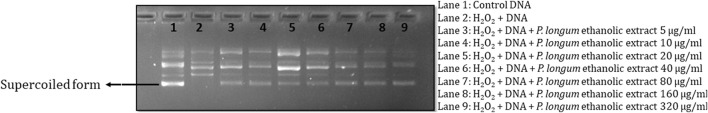
Experimental design to assess the safety and genome-stabilizing action of *Piper longum*.

H_2_O_2_ treatment resulted in a loss of the supercoiled DNA form (Figure 1), while the simultaneous incubation of the *Piper longum* ethanol extract with H_2_O_2_ retained the stable form of DNA, at the concentration range of 5–320 μg/mL.

### 2.2 Protective effects of *Piper longum* against cyclophosphamide-induced cytogenetic damage

As the ethanol extract of *Piper longum* presented a good genoprotective profile in DNA cleavage assay, we further aimed to separate the phytoconstituents and obtained the following fractions: hexane fraction, dichloromethane (DCM) fraction, ethyl acetate fraction, n-butanol fraction, and an aqueous fraction of *Piper longum*. Furthermore, the screening of the most potent fraction possessing genoprotective action was achieved by using a battery of cytogenetic assays (micronucleus assay and chromosomal aberration assay). However, prior to cytogenetic assays, cell viability was examined in order to determine the non-toxic concentration of test chemicals. The extract and fractions were selected in the concentration range of 10–40 μg/mL to perform subsequent analysis, as the cell viability was found to be more than 80% in all tested concentrations.

In the cytokinesis-block micronucleus (CBMN) assay, cyclophosphamide-treated cells show a significant increase in micronucleus (MNi) frequency in comparison to control (Table 1). The simultaneous incubation of the *Piper longum* ethanol extract with cyclophosphamide reduced the MNi in a dose-proportionate fashion, compared to the cells exposed to cyclophosphamide alone. The highest effective concentration of the *Piper longum* ethanol extract was found to be 40 μg/mL, which resulted in a 81.8% reduction of the MNi. Furthermore, amid various fractions (hexane, DCM, ethyl acetate, butanol, and aqueous), which were simultaneously incubated with cyclophosphamide, only the hexane fraction was able to significantly reduce the MNi. The hexane fraction of *Piper longum* at the concentration of 40 μg/mL presented a 75.7% reduction in the MNi.

**TABLE 1:** Evaluation of genetic damage in cultured human lymphocytes treated with *Piper longum* (extract and fractions) and cyclophosphamide in simultaneous incubation experiments.

Test agent	Concentration	Frequency of micronucleated binuclear cells	Percent reduction (%)	Total number of cells with chromosome aberrations
Untreated cells	-	05		04
Cyclophosphamide	5 μg/mL	38***		25***
Ethanol extract + cyclophosphamide	10 μg/mL	18^$^	60.6	12^$^
	20 μg/mL	13^$$$^	75.75	10^$$^
	40 μg/mL	11^$$$^	81.81	08^$$^
Hexane fraction + cyclophosphamide	10 μg/mL	20^$^	54.54	13^$^
	20 μg/mL	18^$^	60.6	13^$^
	40 μg/mL	13^$$$^	75.75	09^$$^
DCM fraction + cyclophosphamide	10 μg/mL	30	24.24	23
	20 μg/mL	30	24.24	21
	40 μg/mL	25	39.39	21
Ethyl acetate fraction + cyclophosphamide	10 μg/mL	32	18.18	24
	20 μg/mL	30	24.24	23
	40 μg/mL	30	24.24	23
Butanol fraction + cyclophosphamide	10 μg/mL	34	12.12	25
	20 μg/mL	32	18.18	24
	40 μg/mL	32	18.18	24
Aqueous fraction + cyclophosphamide	10 μg/mL	38	0	25
	20 μg/mL	36	6.06	25
	40 μg/mL	36	6.06	24

Frequency of micronucleated binuclear cells present the number of binucleated cells having a micronucleus per 1,000 binucleated cells, analyzed by the Fisher’s exact test. The significance was determined by the following:

^$^ *p* < 0.05.

^$$^ *p* < 0.01.

^$$$^ *p* < 0.001 versus cyclophosphamide; ***p < 0.001 versus untreated cells.

The chromosomal aberration (CA) assay was also performed as a test battery to study the protective action of the extract and fraction (Table 1). Cyclophosphamide-treated cells show a significant increment of structural aberrations (chromatid breaks, fragments, and chromosome breaks) in comparison to control. Furthermore, numerical aberrations were not observed in the current experiment. The *Piper longum* ethanol extract and hexane fraction in combination with cyclophosphamide significantly reduced the frequency of aberrations compared to positive control. Similar to the CBMN assay, all other fractions were non-significant when compared to positive control.

### 2.3 Identification of markers (piperine and piperlongumine) by HPTLC

The *Piper longum* ethanol extract and hexane fraction showed genoprotective actions under *in vitro* conditions; hence, the extract and fraction were assessed for the particular markers present in them. The high-performance thin-layer chromatography (HPTLC)-densitometric method was developed and validated to analyze piperine and piperlongumine content in the *Piper longum* extract and hexane fraction. Chromatograms, overlay spectra, and the peak for standards are shown in Supplementary Figure S1. The *Piper longum* ethanol extract contains 0.91% and 0.66% of piperine and piperlongumine, respectively, while the hexane fraction contains 2.91% and 1.83% of piperine and piperlongumine, respectively. The validation parameters for the developed HPTLC method were found to be acceptable (Supplementary Table S1).

### 2.4 Metabolite fingerprinting of the *Piper longum* ethanol extract by UPLC–MS

The prevalence of alkaloids, phenolics, flavonoids, and lignans was identified in the metabolite profile of the *Piper longum* ethanol extract using ultra-high performance liquid chromatography–mass spectroscopy (UPLC-MS). The chromatogram and mass spectra of tentatively identified metabolites are presented in Supplementary Figure S2 and Supplementary Table S2. Metabolites were recognized by retention times, mass by charge ratio (m/z), nature of compounds, and the chemical formula with the respective literature IDs. Some common and major metabolites of *Piper longum* are aristolactam BII (Rt 4.4), isopiperine (Rt 4.7), piperine (Rt 4.7), piperlongumine (Rt 4.4), rosmarinic acid (Rt 4.4), kaempferol (Rt 4.7), and sylvatine (Rt 6.5).

### 2.5 Metabolite fingerprinting of the *Piper longum* hexane fraction by GC-MS

Phytochemical analyses of the hexane fraction of *Piper longum* by the gas chromatography–mass spectrometry (GC-MS) investigation identified approximately 57 compounds, the majority of which belong to the fatty acid and terpenoid classes. The chromatogram and mass spectra of tentatively identified metabolites are presented in Supplementary Figure S3 and Supplementary Table S3. Some common metabolites are limonene (Rt 7.5), linalool (Rt 8.9), octadecanoic acid (Rt 10.3), octanoic acid (Rt 10.7), myristic acid (Rt 18.9), oleic acid (Rt 18.5), palmitic acid (Rt 21.7), and linoleic acid (Rt 25.7).

### 2.6 *In vivo* studies: Safety assessment of the *Piper longum* ethanol extract and hexane fraction of *Piper longum*

Acellular medium and cytogenetic analyses highlight the genomic-stabilizing potential of the extract and fraction; however, these tests do not reveal the underlying mechanisms. Thus, the activity further needs to be validated under the *in vivo* system. Moreover, preceding the genomic instability experiment, it is critical to test for safety assessments as per the Organization for Economic Co-operation and Development (OECD) guidelines. Hence, the *Piper longum* extract and hexane fraction were evaluated for safety assessments by conducting acute (single/fixed dose procedure) and sub-acute toxicity (oral doses repeated for 28 days) studies.

In accordance with the results of the acute oral toxicity study, exposing animals with a single dosage of the *Piper longum* ethanol extract and hexane fraction (2000 mg/kg) is not associated with physiological or behavioral changes, along with no deaths during the course of the 14-day period. The macroscopic examination of organs revealed no abnormal lesions. Additionally, neither hematology nor biochemical analyses showed any clinically important variations from the control group (Supplementary Table S4, 5).

During the sub-acute toxicity protocol, the *Piper longum* ethanolic extract and hexane fraction at the dose of 200, 400, and 800 mg/kg for a 28-day administration period demonstrated no toxic signs and no mortalities were logged. Hematological indices also demonstrated the standard physiological range for all groups (Supplementary Table S6, 7). With reference to hepatic function parameters, the oral administration of the extract and fraction did not cause any significant changes except for the 800 mg/kg dose; the hexane fraction of *Piper longum* showed a substantial increase in ALP, AST, and ALT. Furthermore, the levels of bilirubin, urea, uric acid, creatinine, protein, calcium, and glucose of rats were observed to be within standard biological limits. There were no significant alterations in the lipid profile; only male rats administered with *Piper longum* (ethanolic extract and hexane fraction) presented a significant rise in the levels of HDL-C 200 mg/kg (Table 2). Furthermore, substantial alterations in animal groups during the recovery period was not detected (Supplementary Table S8, 9).

**TABLE 2:** Serum biochemistry analyses of rats administered with the *Piper longum* ethanolic extract and hexane fraction for 28 days.

S. No	Treatment group	Control	PLE 200 mg/kg	PLE 400 mg/kg	PLE 800 mg/kg	PLE-H 200 mg/kg	PLE-H 400 mg/kg	PLE-H 800 mg/kg
	**Male**							
**1**	Alkaline phosphatase (U/I)	124.0 ± 9.03	106.8 ± 8.47*	109 ± 9.81	112.5 ± 9.86	109.3 ± 9.44	111.8 ± 10.73	144.2 ± 10.5*
**2**	Aspartate aminotransferase (U/L)	110.2 ± 7.27	101 ± 6.02*	122.7 ± 6.53*	122.7 ± 8.17	111.7 ± 5.31	123.1 ± 3.37*	126.8 ± 5.93**
**3**	Alanine aminotransferase (U/I)	40.93 ± 3.11	37.58 ± 3.29	40.32 ± 3.68	41.84 ± 4.10	37.03 ± 2.94	47.37 ± 2.39*	48.59 ± 3.13**
**4**	Bilirubin total (mg/dL)	00.42 ± 0.06	00.34 ± 0.04	00.38 ± 0.05	00.38 ± 0.05	00.42 ± 0.03	00.45 ± 0.03	00.38 ± 0.049
**5**	Bilirubin direct (mg/dL)	0.08 ± 0.008	0.073 ± 0.006	0.079 ± 0.008	0.075 ± 0.006	0.082 ± 0.007	0.075 ± 0.006	0.078 ± 0.008
**6**	Blood urea nitrogen (mg/dL)	42.90 ± 2.59	43.97 ± 2.36	42.41 ± 2.44	45.85 ± 3.49	44.61 ± 1.21	46.65 ± 2.79	46.92 ± 2.93
**7**	Uric acid (mg/dL)	2.09 ± 0.10	2.37 ± 0.36	2.28 ± 0.17	2.51 ± 0.15*	1.96 ± 0.13	2.10 ± 1.98	2.38 ± 0.28
**8**	Creatinine (mg/dL)	0.59 ± 0.057	0.57 ± 0.059	0.56 ± 0.051	0.57 ± 0.056	0.59 ± 0.052	0.58 ± 0.035	0.57 ± 0.051
**9**	Total protein (g/dL)	6.93 ± 0.29	7.26 ± 0.27	6.89 ± 0.33	7.41 ± 0.31	7.34 ± 0.31	7.19 ± 0.32	7.30 ± 0.40
**10**	Albumin (g/dL)	3.04 ± 0.19	3.36 ± 0.20	3.02 ± 0.30	3.43 ± 0.3*	3.28 ± 0.17	3.20 ± 0.23	2.94 ± 0.11
**11**	Calcium (mg/dL)	9.34 ± 0.41	9.11 ± 0.62	9.00 ± 0.52	10.21 ± 0.73	9.25 ± 0.38	9.13 ± 0.54	9.88 ± 0.52
**12**	Glucose (mg/dL)	109.3 ± 12.52	107.4 ± 10.87	111 ± 12.11	110.2 ± 10.2	108.8 ± 7.77	107.5 ± 11.16	111 ± 12.12
**13**	Total cholesterol (mg/dL)	66.63 ± 3.50	60.72 ± 3.37	68.58 ± 3.72	70.69 ± 3.78	63.57 ± 3.98	65.63 ± 4.13	68.33 ± 4.68
**14**	Triglycerides (mg/dL)	34.77 ± 1.92	32.86 ± 1.65	35.16 ± 2.11	35.84 ± 2.86	33.96 ± 2.69	36.03 ± 1.84	34.23 ± 2.67
**15**	High density lipoprotein (mg/dL)	61.24 ± 2.49	66.38 ± 3.19*	63.25 ± 2.95	64.84 ± 3.58	67.30 ± 2.71*	65.70 ± 3.34	65.18 ± 1.91
**16**	Very low density lipoprotein (mg/dL)	5.77 ± 0.31	5.45 ± 0.27	5.83 ± 0.35	5.94 ± 0.47	5.63 ± 0.44	5.98 ± 0.30	5.68 ± 0.44
	**Female**							
**1**	Alkaline phosphatase (U/I)	131.4 ± 11.17	119.1 ± 12.80	122.2 ± 10.02	123.3 ± 9.10**	122.6 ± 11.8	123.6 ± 10.80	158.2 ± 7.71**
**2**	Aspartate aminotransferase (U/L)	112.2 ± 6.86	105.5 ± 6.29	126.9 ± 5.09*	128.1 ± 2.00**	113.4 ± 8.81	126.4 ± 6.34*	129.4 ± 3.56**
**3**	Alanine aminotransferase (U/I)	35.08 ± 3.61	33.91 ± 3.68	37.79 ± 4.27	38.36 ± 3.75	35.30 ± 3.30	40.49 ± 2.97	45.07 ± 3.58**
**4**	Bilirubin total (mg/dL)	0.31 ± 0.046	0.28 ± 0.043	0.27 ± 0.20	0.25 ± 0.038	0.32 ± 0.034	0.32 ± 0.031	0.37 ± 0.047
**5**	Bilirubin direct (mg/dL)	0.10 ± 0.008	0.10 ± 0.007	0.10 ± 0.009	0.09 ± 0.007	0.10 ± 0.01	0.11 ± 0.016	0.09 ± 0.007
**6**	Blood urea nitrogen (mg/dL)	44.23 ± 2.58	44.31 ± 2.94	46.46 ± 1.62	47.66 ± 2.11	43.86 ± 1.82	46.72 ± 1.24	48.25 ± 3.99
**7**	Uric acid (mg/dL)	1.82 ± 0.15	1.95 ± 0.11	1.94 ± 0.16	2.08 ± 0.21	1.71 ± 0.16	1.70 ± 0.16	2.05 ± 0.11
**8**	Creatinine (mg/dL)	0.45 ± 0.046	0.43 ± 0.065	0.50 ± 0.058	0.45 ± 0.042	0.45 ± 0.043	0.41 ± 0.051	0.41 ± 0.034
**9**	Total protein (g/dL)	6.84 ± 0.42	6.61 ± 0.32	6.95 ± 0.34	6.95 ± 0.42	6.50 ± 0.36	6.64 ± 0.32	6.83 ± 0.38
**10**	Albumin (g/dL)	3.27 ± 0.16	3.45 ± 0.19	3.10 ± 0.27	3.06 ± 0.13	3.58 ± 0.23	3.51 ± 0.34	3.38 ± 0.23
**11**	Calcium (mg/dL)	8.89 ± 0.71	8.63 ± 0.40	9.01 ± 0.29	9.12 ± 0.44	8.96 ± 0.54	9.14 ± 0.51	9.22 ± 0.34
**12**	Glucose (mg/dL)	116.1 ± 10.6	118.17 ± 12.7	114.6 ± 12.17	115.81 ± 12.6	117.12 ± 12.5	115.16 ± 11.4	117.88 ± 10.4
**13**	Total cholesterol (mg/dL)	65.13 ± 3.47	61.51 ± 4.69	67.68 ± 3.85	70.86 ± 5.11	62.07 ± 3.47	67.41 ± 3.85	68.29 ± 4.13
**14**	Triglycerides (mg/dL)	38.89 ± 2.00	35.05 ± 1.99	37.86 ± 2.38	38.07 ± 2.12	36.87 ± 2.53	40.27 ± 2.98	39.19 ± 3.27
**15**	High density lipoprotein (mg/dL)	63.89 ± 2.33	67.92 ± 2.69	66.17 ± 1.92	64.24 ± 3.15	67.26 ± 1.31	65.58 ± 4.06	63.08 ± 2.60
**16**	Very low density lipoprotein (mg/dL)	6.45 ± 0.33	5.81 ± 0.33	6.28 ± 0.39	6.31 ± 0.35	6.12 ± 0.42	6.68 ± 0.49	6.50 ± 0.54

Data are presented as mean ± SD (*n* = 5 per group) and analyzed by the one-way analysis of variance followed by Dunnett’s test. PLE, *Piper longum* ethanolic extract; PLE-H, hexane fraction of *Piper longum*. Significance was determined as *p < 0.05 and **p < 0.01 versus the control group.

### 2.7 Effects of *Piper longum* on a cyclophosphamide-induced model of genomic instability

#### 2.7.1 Body weight

**FIGURE 2:**
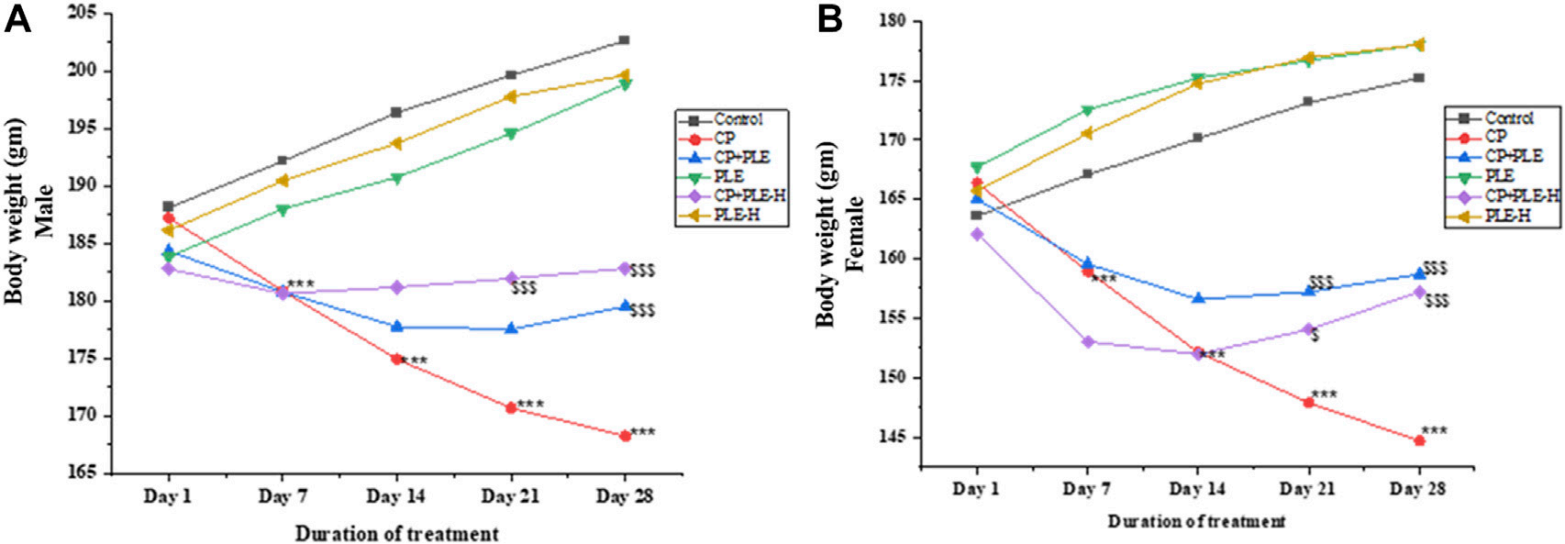
Cell-free medium: protective effect of the *Piper longum* ethanol extract on DNA strand breaks against H_2_O_2_-induced genome damage.

During the 28-day treatment, body weight changes were recorded on a weekly basis (Figure 2). Control animals presented a gain in body weight, while cyclophosphamide-treated rats showed a significant (*p* < 0.001) decline in body weight from day 7 onward. It was further observed that the extract and fraction co-treated with cyclophosphamide resisted body weight loss from day 14 onward and plateaued for the remaining duration of the study. On the 28th day, the fraction and extract of *Piper longum* co-treated with cyclophosphamide showed a significant increment in body weight (*p* < 0.001) when compared to the positive control.

#### 2.7.2 Organ indexes (terminal organ weight)

**FIGURE 3:**
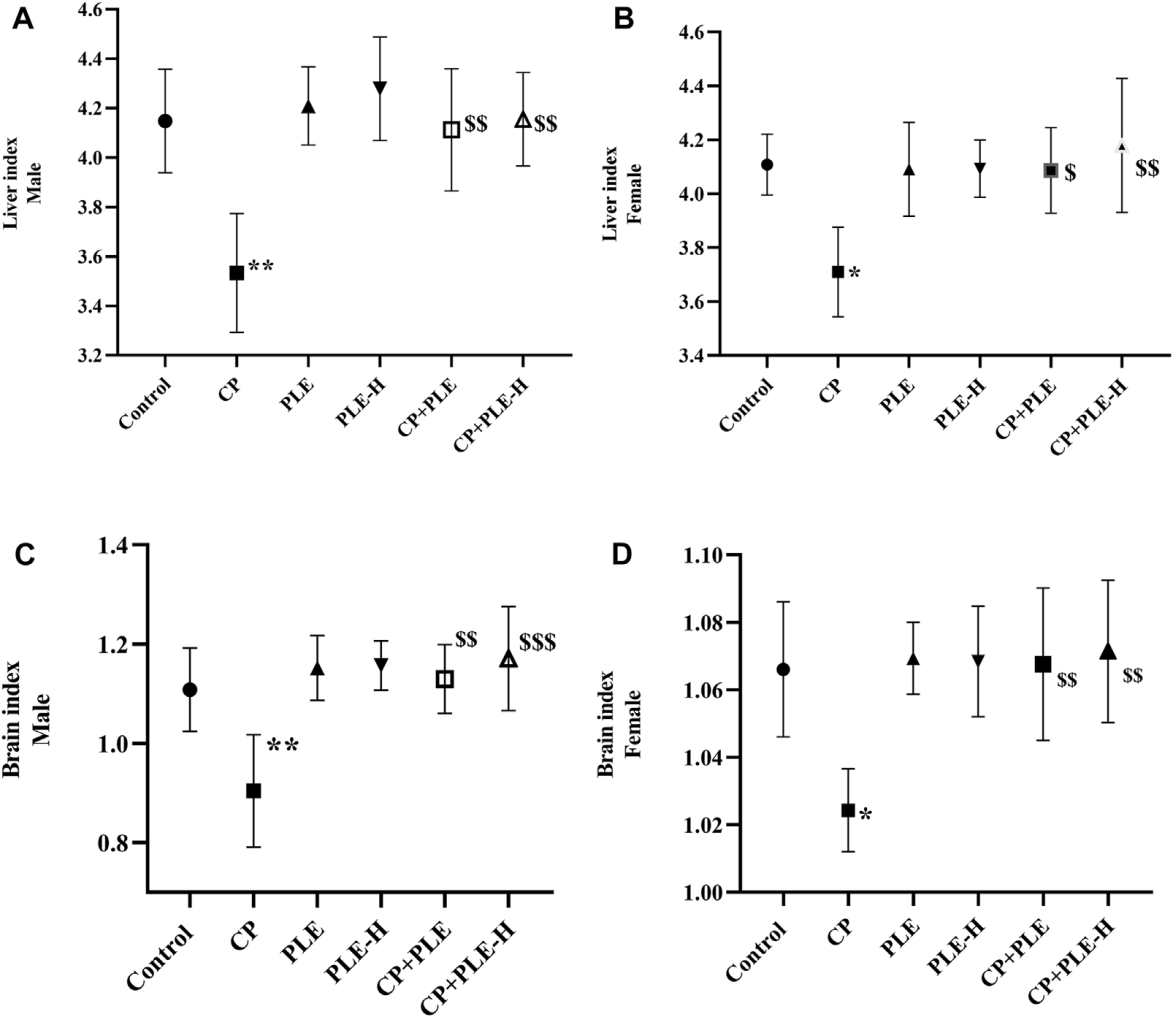
Changes in the body weight of rats administered with the *Piper longum* ethanol extract and hexane fraction, alone or in combination with cyclophosphamide for 28 days. **(A)** Body weight changes in Wistar albino male rats. **(B)** Body weight changes in Wistar albino female rats. The values are expressed as mean ± SD (*n* = 5 per group). The one-way ANOVA followed by Tukey’s multiple-comparison test was performed. *** = *p* < 0.001, significant when compared with the normal control group; ^$^ = *p* < 0.05, ^$$^ = *p* < 0.01, and ^$$$^ = *p* < 0.001, significant when compared with the CP-treated group. PLE = *Piper longum* ethanolic extract: 200 mg/kg/day; PLE-H = *Piper longum* hexane fraction: 200 mg/kg/day; CP = cyclophosphamide: 5 mg/kg/day.

The relative organ weight of the liver and brain are presented in Figure 3. A cyclophosphamide-induced sharp decrement of the liver index in both genders was seen, while the co-treatment of the extract and fraction significantly (*p* < 0.01) restored the liver index. Likewise, the brain index was lower in cyclophosphamide-treated rats than that in negative control, whereas the co-treatment of the extract and fraction resulted in a reversal of the brain index toward negative control rats.

#### 2.7.3 *In vivo* bone marrow micronuclei assays

Cyclophosphamide administration caused a considerable elevation (*p* < 0.001) of the micronucleus and a decline in PCE/NCE ratios. The *Piper longum* ethanol extract suppressed this generation by 28.5% in male rats and 27.09% in female animals. Additionally, the hexane fraction of *Piper longum* caused a 33.33% decrease in males and a 28.3% decrease in the female group. Additionally, the cytotoxicity was also reversed by test agents (Table 3).

**TABLE 3:** Results of the micronucleus test after the administration of the *Piper longum* extract.

Group	Micronucleated polychromatic erythrocytes	Percent reduction (%)	Micronucleated polychromatic erythrocytes	Percent reduction (%)	Ratio of polychromatic erythrocytes to normochromatic erythrocytes	
Male		Female			Male	Female
Control	7		9		1.05 ± 0.10	1.08 ± 0.17
Cyclophosphamide (CP)	154***		164***		0.48 ± 0.05***	0.42 ± 0.04***
PLE	6		7		1.44 ± 0.37	1.37 ± 0.31
PLE-H	6		6		1.49 ± 0.26	1.41 ± 0.29
CP + PLE	112^$$^	28.57	122^$^	27.09	0.98 ± 0.19^$^	0.97 ± 0.19^$$^
CP + PLE-H	105^$$^	33.33	120^$$^	28.38	0.98 ± 0.22^$^	0.99 ± 0.19^$$^

Frequency of micronucleated polychromatic erythrocytes was statistical analyzed by the chi-square test. The ratio of polychromatic erythrocytes to normochromatic erythrocytes is presented as mean ± SD (*n* = 5 per group) and analyzed by the one-way analysis of variance followed by Tukey’s multiple-comparison test. The significance was determined by ***p < 0.001 versus the control;

^$$^ *p* < 0.05 versus CP;

^$^ *p* < 0.01 versus CP. PLE = *Piper longum* ethanolic extract (200 mg/kg/day) and PLE-H hexane fraction of *Piper longum* (200 mg/kg/day).

#### 2.7.4 Diphenylamine method for evaluating DNA fragmentation

**FIGURE 4:**
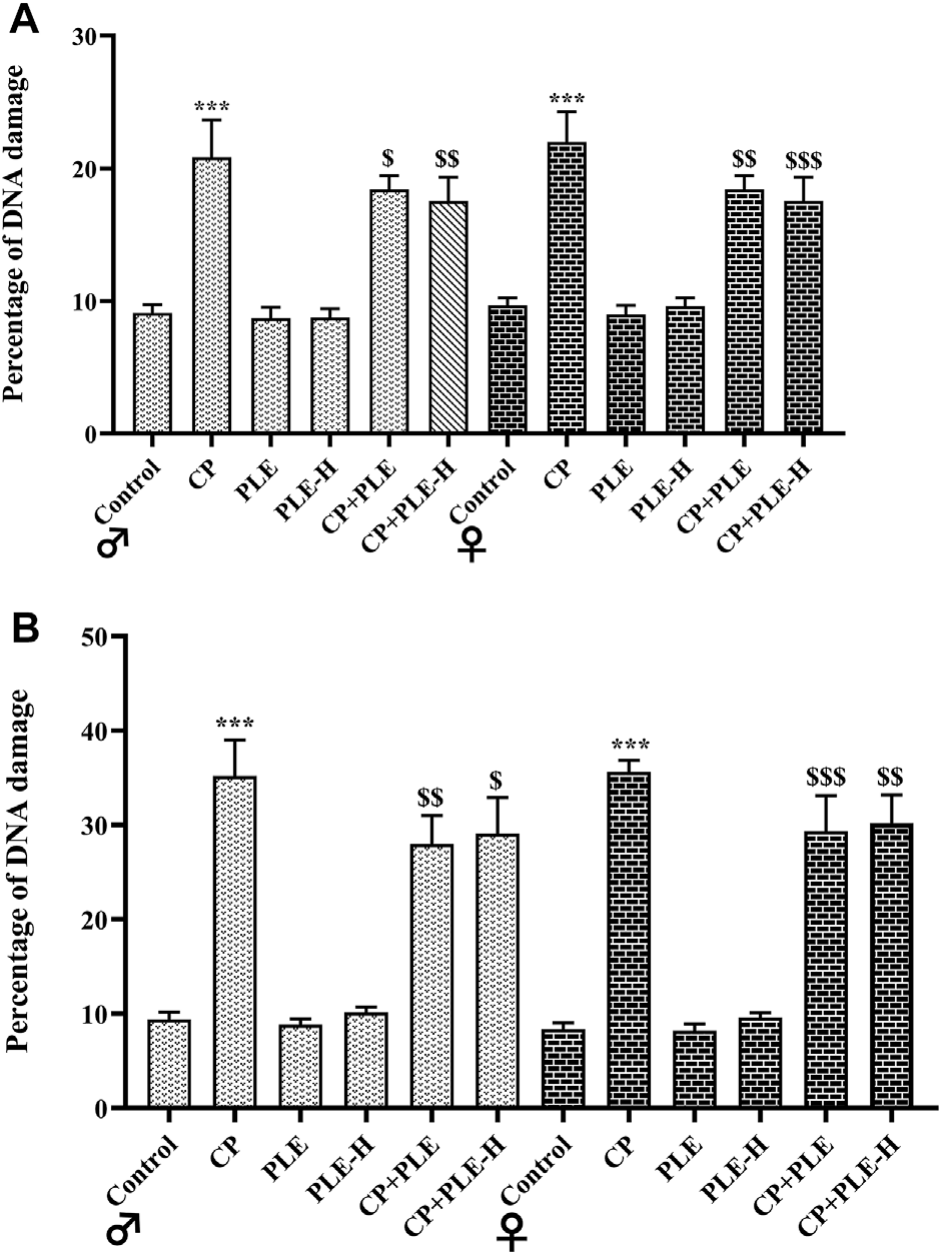
Liver and brain index of rats administered with the *Piper longum* ethanol extract and hexane fraction, alone or in combination with cyclophosphamide for 28 days. **(A, B)** Liver index of male and female Wistar albino rats. **(C, D)** Brain index of male and female Wistar albino rats. The values are expressed as mean ± SD (*n* = 5 per group). The one-way ANOVA followed by Tukey’s multiple-comparison test was performed. * = significantly different versus the control group (*p* < 0.05); ^$^ = significantly different versus the CP-treated group (*p* < 0.05); ^$$^ = significantly different versus the CP-treated group (*p* < 0.01). PLE = *Piper longum* ethanolic extract: 200 mg/kg/day; PLE-H = *Piper longum* hexane fraction: 200 mg/kg/day; CP = cyclophosphamide: 5 mg/kg/day.

Cyclophosphamide treatment resulted in DNA fragmentation, with more fragmentation in the hippocampus (Male: 25.78%; female: 27.22%) than the hepatic tissue (Male: 11.47%; female: 12.31%) (*p* < 0.001). However, the simultaneous administration of test agents with toxicants resulted in a slight reduction of DNA fragmentation, with the *Piper longum* ethanol extract showing better effects on brain tissue, while the hexane fraction showed better effects on the liver (Figure 4).

#### 2.7.5 Free-radical generation, MDA, and GSH levels

**FIGURE 5:**
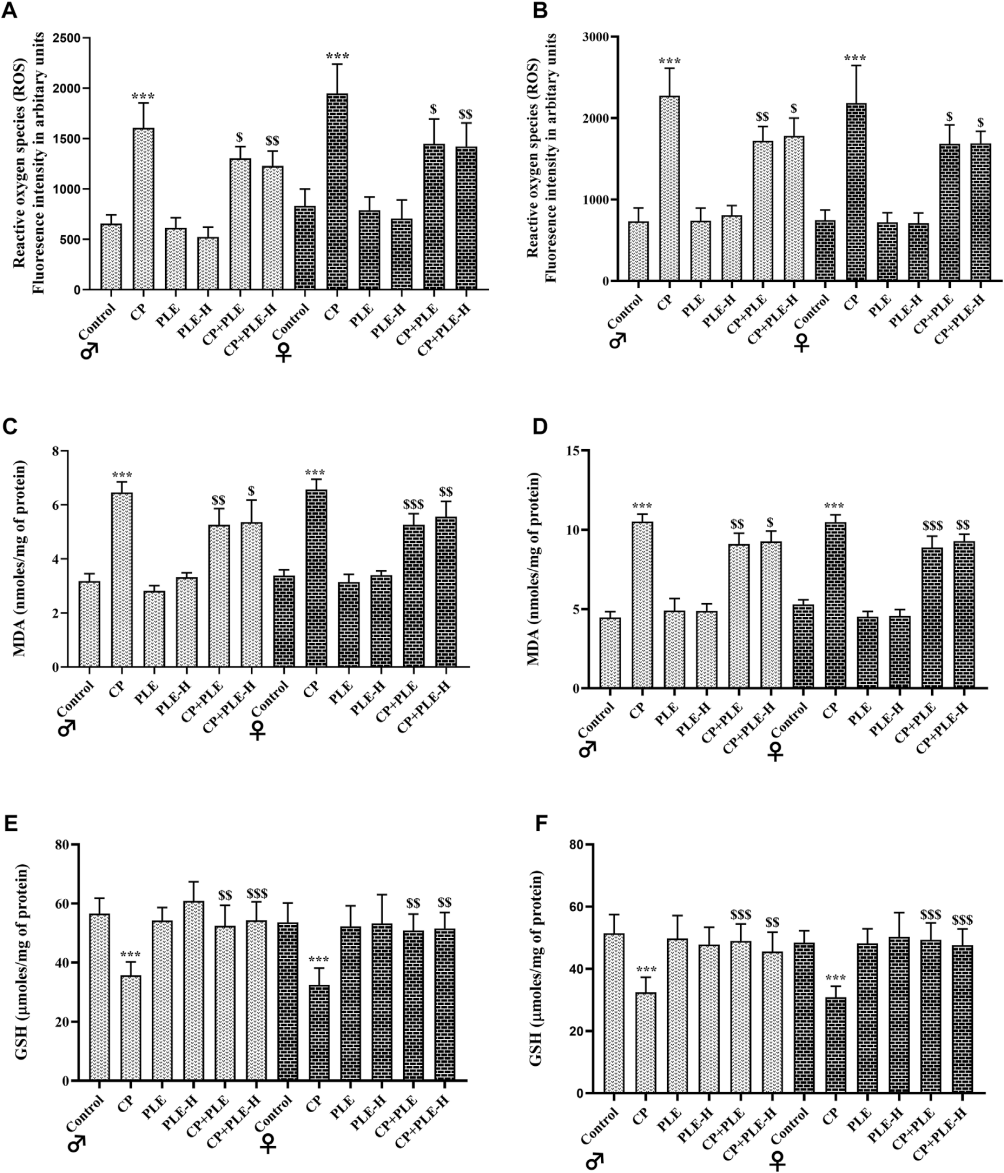
Influence of the *Piper longum* ethanol extract and the *Piper longum* hexane fraction on the DNA fragmentation percentage of cyclophosphamide-intoxicated rats. **(A)** Percentage DNA fragmentation in hepatic tissue of rats from both genders. **(B)** Percentage DNA fragmentation in the hippocampus of rats from both genders. Data are presented as mean ± SD (*n* = 5 per group) and analyzed by the one-way analysis of variance followed by Tukey’s multiple-comparison test. The significance was established by ***p < 0.001 versus the control; ^$$^ *p* < 0.05 and ^$^ *p* < 0.01 versus CP. PLE = *Piper longum* ethanolic extract: 200 mg/kg/day; PLE-H = *Piper longum* hexane fraction: 200 mg/kg/day; CP = cyclophosphamide: 5 mg/kg/day.

In hepatic and hippocampus tissue, cyclophosphamide treatment reduced glutathione (GSH), while increasing the malondialdehyde (MDA) concentration and free-radical production (*p* < 0.001). The simultaneous administration of the *Piper longum* extract and hexane fraction with cyclophosphamide reversed the levels of reactive oxygen species (ROS) and GSH toward negative control; however, the levels of MDA were only slightly averted by test agents (Figure 5).

#### 2.7.6 Estimation of 8-OHdG

**FIGURE 6:**
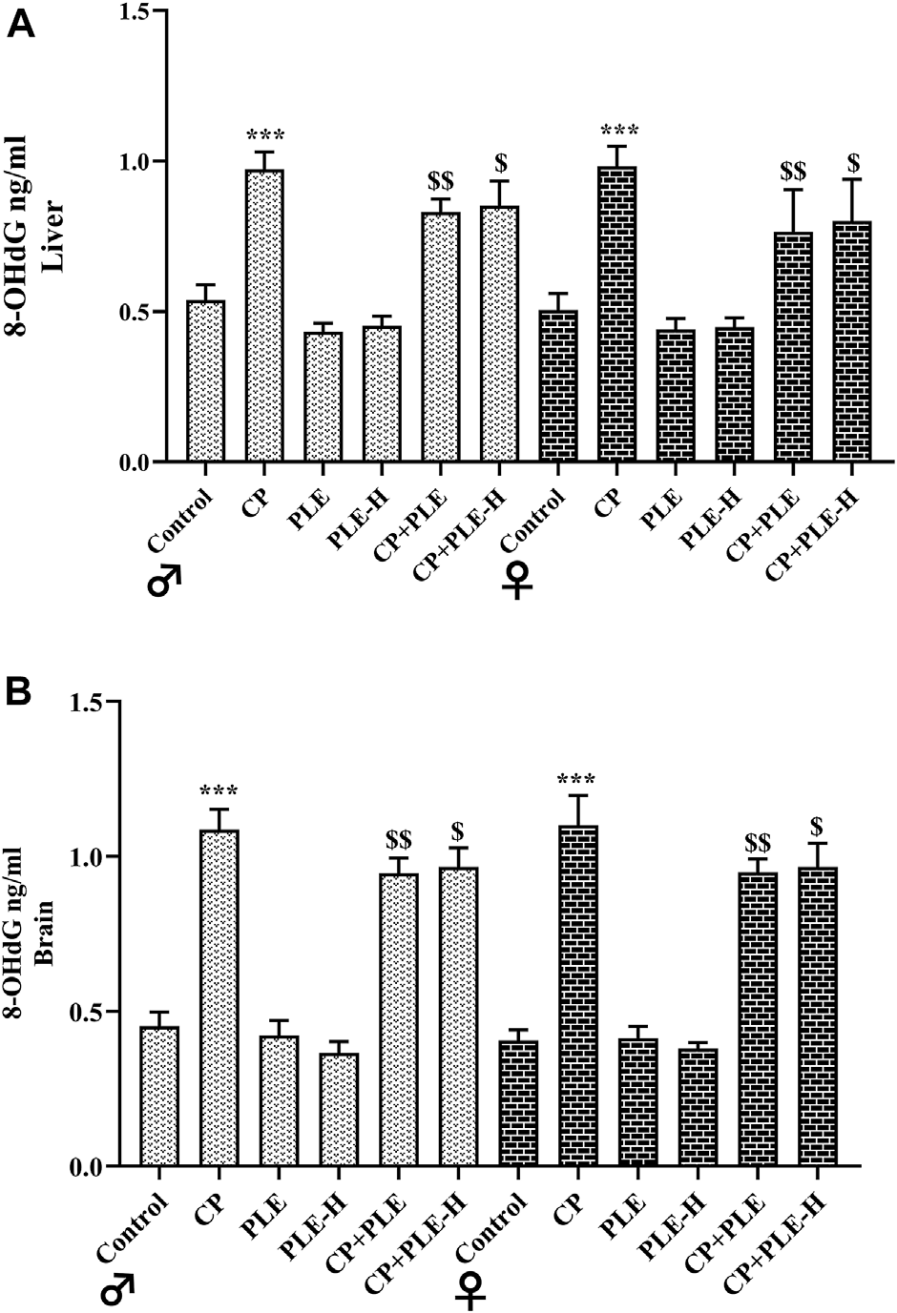
Influence of the *Piper longum* ethanol extract and *Piper longum* hexane fraction on reactive oxygen species (ROS), malondialdehyde (MDA), and glutathione (GSH) levels of cyclophosphamide-intoxicated rats. **(A)** ROS generation in hepatic tissue of rats from both genders. **(B)** ROS generation in the hippocampus from rats of both genders. **(C)** MDA levels in hepatic tissue from rats of both genders. **(D)** MDA levels in hippocampus tissue from rats of both genders. **(E)** GSH levels in hepatic tissue from rats of both genders. **(F)** GSH levels in the hippocampus tissue from rats of both genders. Data are presented as mean ± SD (*n* = 5 per group) and analyzed by one-way analysis of variance followed by Tukey’s multiple-comparison test. The significance was established by ***p < 0.001 versus the control; ^$$^ *p* < 0.05 and ^$^ *p* < 0.01 versus CP. PLE = *Piper longum* ethanolic extract: 200 mg/kg/day; PLE-H = *Piper longum* hexane fraction: 200 mg/kg/day; CP = cyclophosphamide: 5 mg/kg/day.

8-Hydroxy-2-deoxyguanosine (8-OHdG) was calculated in liver and hippocampus tissue homogenates (Figure 6). Both the tissues presented elevated levels of 8-OHdG upon cyclophosphamide exposure (*p* < 0.001). The treatment with ethanolic extracts and hexane fractions of *Piper longum* resulted in a reduction of the oxidation-induced genome damage biomarker, with the extract showing better effects.

#### 2.7.7 Histopathological findings

##### 2.7.7.1 Liver

**FIGURE 7:**
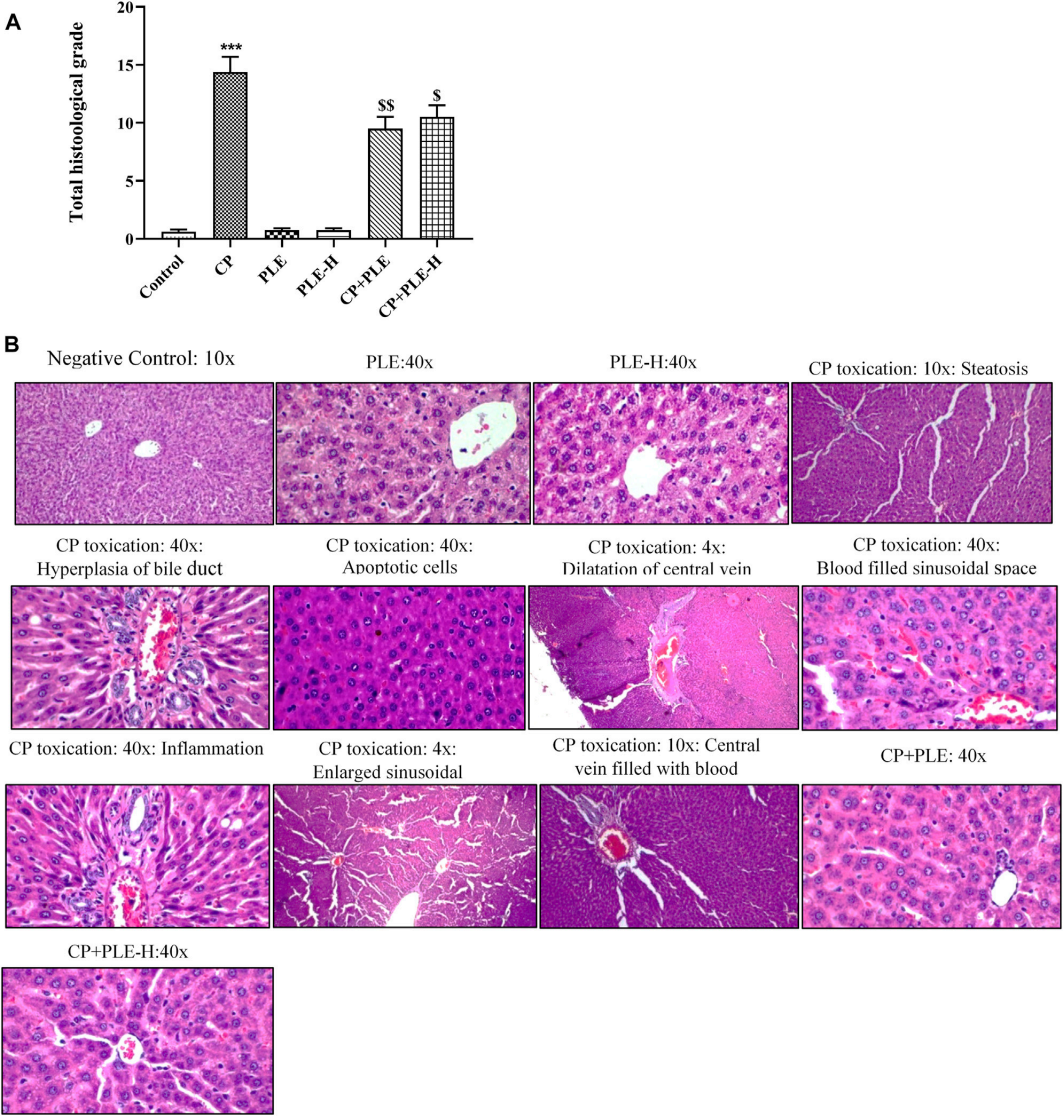
Influence of the *Piper longum* ethanol extract and the *Piper longum* hexane fraction on 8-OHdG levels of cyclophosphamide-intoxicated rats. **(A)** 8-OHdG level in hepatic tissue of rats from both genders. **(B)** 8-OHdG level in hippocampus tissue of rats from both genders. Data are presented as mean ± SD (*n* = 5 per group) and analyzed by one-way analysis of variance followed by Tukey’s multiple-comparison test. The significance was established by ***p < 0.001 versu*s* control; ^$$^ *p* < 0.05 and ^$^ *p* < 0.01 versus CP. PLE = *Piper longum* ethanolic extract: 200 mg/kg/day; PLE-H = *Piper longum* hexane fraction: 200 mg/kg/day; CP = cyclophosphamide: 5 mg/kg/day.

The histological architecture of control rats showed well-organized hepatocytes (Figure 7B). The rats treated with cyclophosphamide show intensive injury in the hepatic tissue counting a large number of apoptotic cells and steatosis, which are described by transparent void vacuoles in the cytoplasm. Although the congestion in the central vein was mild, the dilation of the central vein was intensively observed. Cyclophosphamide treatment further caused intensive sinusoidal dilation, hyperplasia of the bile duct, and infiltration of inflammatory cells. Tissue sections also showed hyperemia and blood-filled sinusoidal spaces in cyclophosphamide-treated groups. Animals intoxicated with cyclophosphamide further presented a substantial rise (*p* < 0.001) in the histology score (Figure 7A), while it was considerably reduced with co-exposure to *Piper longum* (ethanol extract *p* < 0.01 and hexane fraction *p* < 0.05).

##### 2.7.7.2 Hippocampus

**FIGURE 8:**
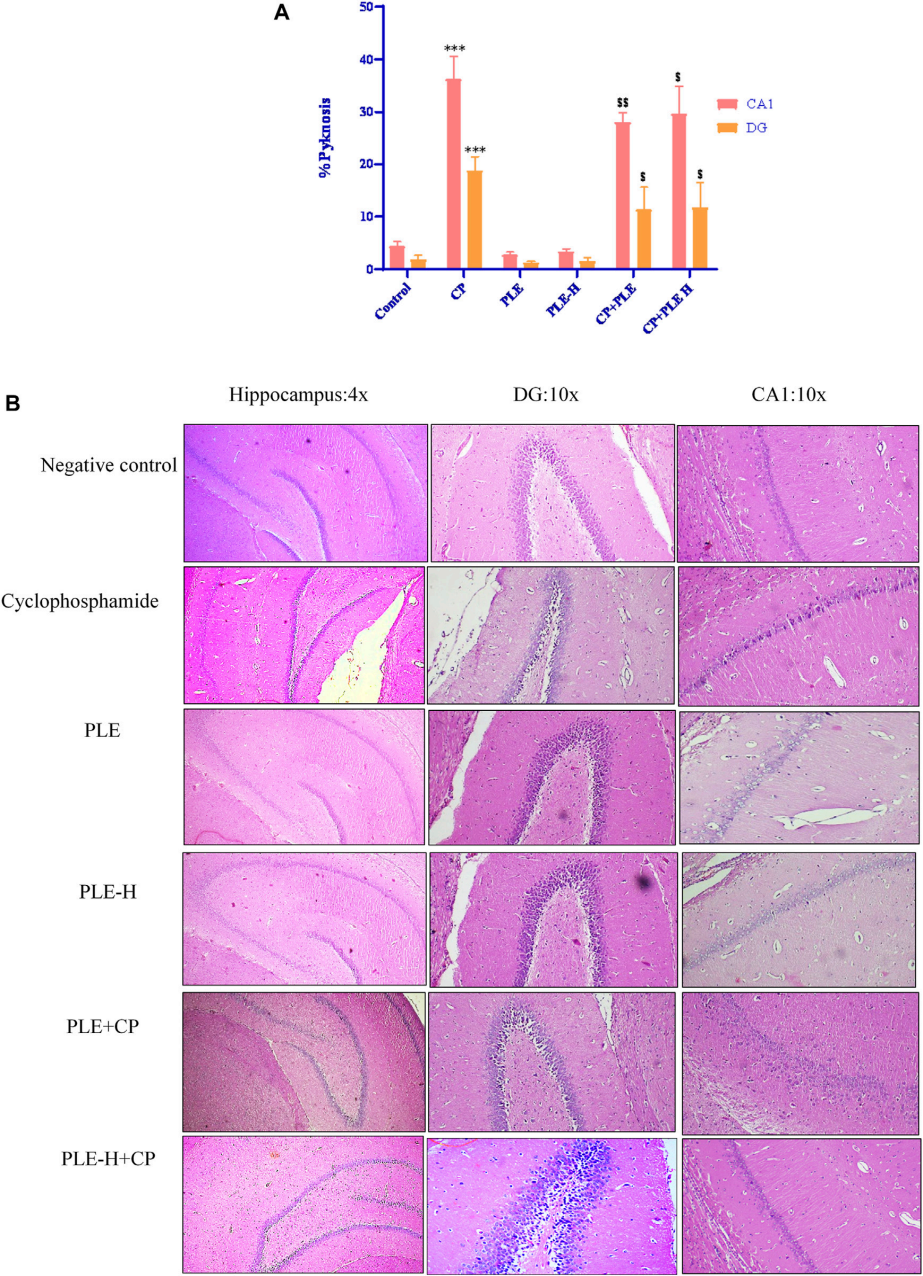
Influence of the *Piper longum* ethanol extract and the *Piper longum* hexane fraction on the histological examination of hepatic tissue in cyclophosphamide-intoxicated rats. **(A)** Total histological grade of hepatic tissue. **(B)** Representative image of hepatic tissue. Data are presented as mean ± SD (*n* = 5 per group) and analyzed by the one-way analysis of variance followed by Tukey’s multiple-comparison test. The significance was established by ***p < 0.001 versus control; ^$$^ *p* < 0.05 and ^$^ *p* < 0.01 versus CP. PLE = *Piper longum* ethanolic extract: 200 mg/kg/day; PLE-H = *Piper longum* hexane fraction: 200 mg/kg/day; CP = cyclophosphamide: 5 mg/kg/day.

In the control group, histological analysis of the hippocampus revealed a normal hippocampal anatomy. Hippocampus slices of cyclophosphamide-intoxicated animals demonstrated abundant tissue damage such as injured neurons, thinning of the neuronal layer, degeneration of pyramidal cells, and nuclei were pyknotic and hyperchromatic (Figure 8B). Cyclophosphamide treatment resulted in severe pyknosis in cornu ammonis 1 (CA1) and in the dentate gyrus (DG) sub-region of the hippocampus (*p* < 0.001) (Figure 8A). In the CA1 sub-region, the *Piper longum* ethanol extract + cyclophosphamide caused a considerable (*p* < 0.01) reduction of pyknosis, followed by the *Piper longum* hexane fraction + cyclophosphamide (*p* < 0.05), while in the DG region, pyknosis reversal was equally affected by the extract and fraction (*p* < 0.05).

#### 2.7.8 Estimation of γH2AX by IHC

**FIGURE 9:**
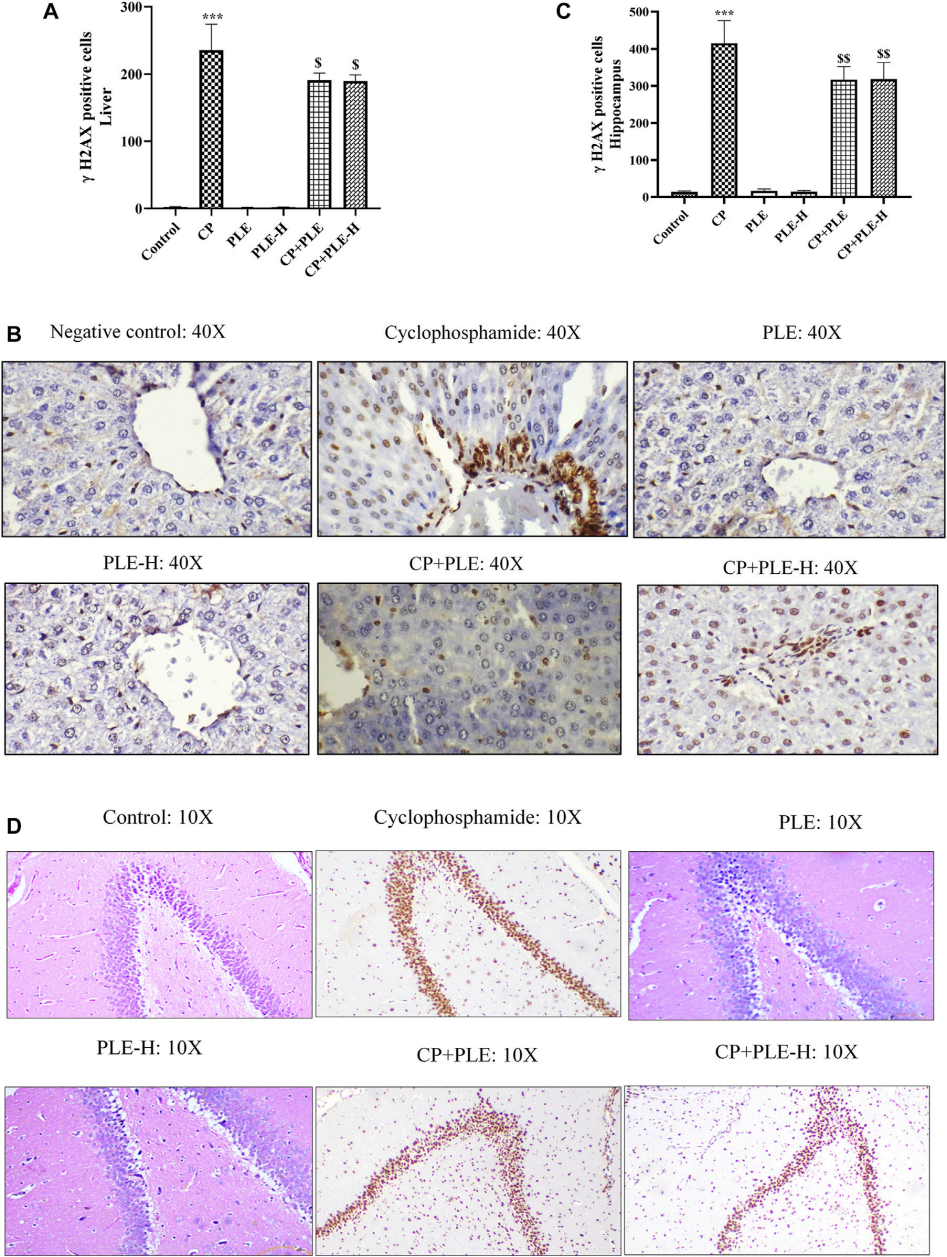
Influence of the *Piper longum* ethanol extract and the *Piper longum* hexane fraction on the histological examination of the hippocampus tissue in cyclophosphamide-intoxicated rats. **(A)** Histopathological assessment of the hippocampus in different experimental groups represented by percentage pyknosis. **(B)** Representative photomicrographs of hippocampal sections under lower magnification (×4) and corresponding sub-sections (CA1 and DG) under higher magnification (×10). Data are presented as mean ± SD (*n* = 5 per group) and analyzed by one-way analysis of variance followed by Tukey’s multiple-comparison test. The significance was established by ***p < 0.001 versus the control; ^$$^ *p* < 0.05 and ^$^ *p* < 0.01 versus CP. PLE = *Piper longum* ethanolic extract: 200 mg/kg/day; PLE-H = *Piper longum* hexane fraction: 200 mg/kg/day; CP = cyclophosphamide: 5 mg/kg/day.

γH2AX immune-expression was identified in hepatic and hippocampus cells. In both the tissues, γH2AX immune-expression was substantially increased (*p* < 0.001) after cyclophosphamide intoxication, while immunopositivity was reduced in the extract (*p* < 0.01) and fraction (*p* < 0.05) of the exposed animals (Figure 9).

## 3 Materials and methods

### 3.1 Chemicals

Cytochalasin B, 2′,7′-dichlorofluorescin diacetate, and RPMI media were procured from Sigma-Aldrich. KaryoMAX™ Colcemid™ and penicillin–streptomycin were obtained from Thermo Fisher Scientific; cyclophosphamide, from TCI Chemicals (India), while all other chemicals such as o-phthalaldehyde, ethanol, hexane, dichrolomethane, ethyl acetate, n-butanol, and May–Grunwald–Giemsa stain were procured from Sisco Research Laboratories (SRL) Pvt. Ltd., India.

### 3.2 Plant material and extract preparation

Fruits of *Piper longum* were procured from the crude drug market of New Delhi. Crude materials were authenticated following the procedure mentioned in the Ayurvedic Pharmacopeia of India. A voucher specimen (BNPL/JH/Ph.D./10/2019/02) is preserved at the Bioactive Natural Product Laboratory (BNPL), Jamia Hamdard, New Delhi, for future reference. To obtain the ethanolic extract of *Piper longum*, shade-dried fruits were pulverized and extracted with 70% ethanol under reflux. The extract was dried under reduced pressure and stored at 4°C until further use.

### 3.3 DNA strand-break analyses in the plasmid DNA (cell-free medium)

This assay was adopted from studies by Biso et al. (2010) and Chatti et al. (2011). Briefly, pBluescript KS vector plasmids were isolated from DH5α *E. coli* cells using QIAamp DNA Kits according to the manufacturer’s instructions. Plasmid DNA was than treated with H_2_O_2_ alone (20 mM) and co-treated with different concentrations of the *Piper longum* extract for 60 min at 37°C. All the samples were electrophoresed on 1% agarose gel and photographed using the Bio-Rad gel documentation system.

### 3.4 Sequential fractionation

With the aim to delineate and segregate bioactive molecules in the *Piper longum* ethanolic extract, sequential fractionation was achieved by adding solvents with increasing polarities. The following solvents were used: hexane, dichloromethane, ethyl acetate, n-butanol, and water.

### 3.5 Cytogenetic analyses (*in vitro* studies)

**FIGURE 10:**
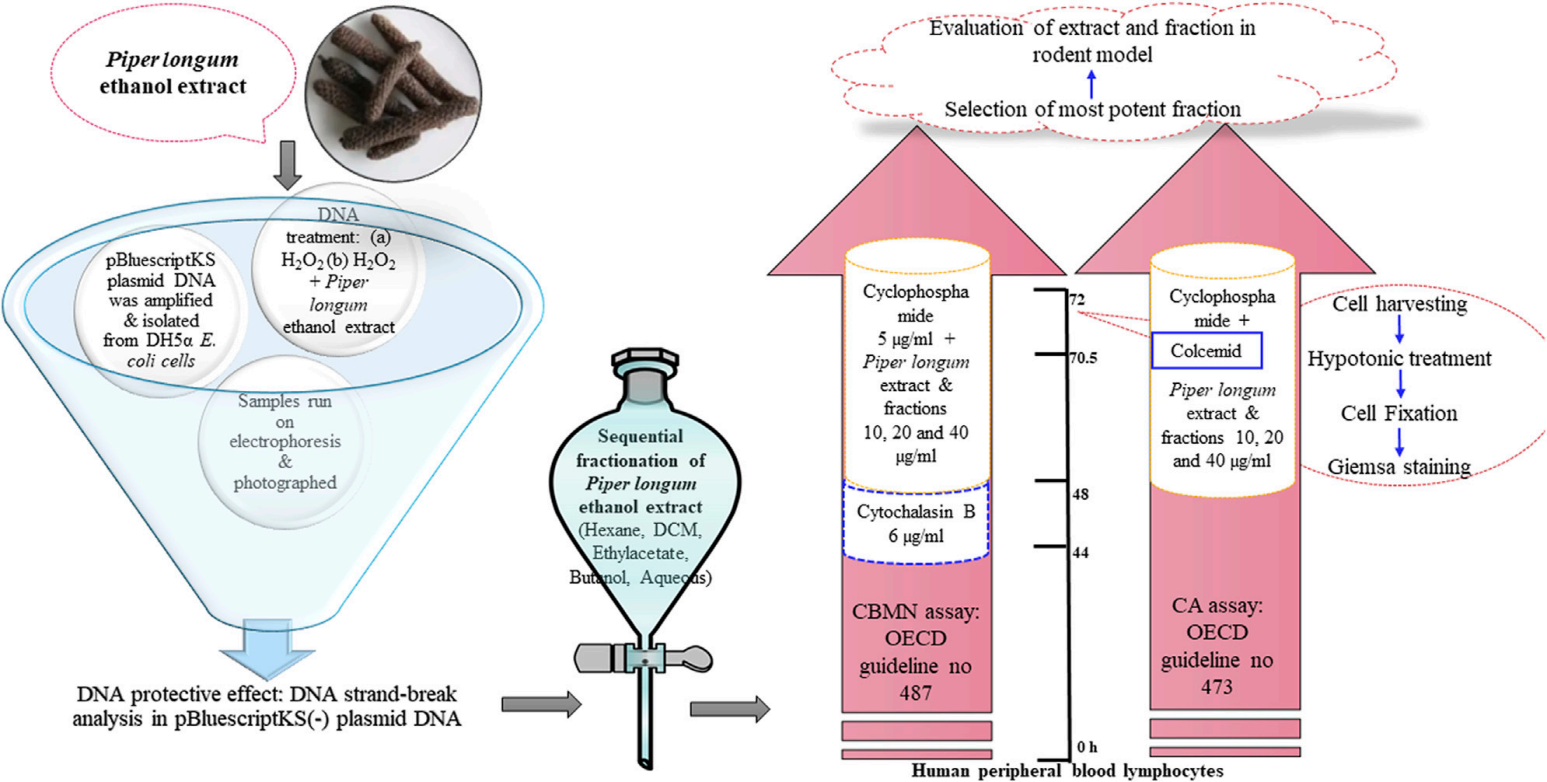
Influence of the *Piper longum* ethanol extract and the *Piper longum* hexane fraction on the γH2AX immune-expression in cyclophosphamide-intoxicated rats. **(A)** Quantitative analysis of hepatic tissue. **(B)** Representative immunohistochemical expression of γH2AX in hepatic tissue. **(C)** Quantitative analysis of the hippocampus. **(D)** Representative immunohistochemical expression of γH2AX in the hippocampus. Data are presented as mean ± SD (*n* = 5 per group) and analyzed by one-way analysis of variance followed by Tukey’s multiple-comparison test. The significance was established by ***p < 0.001 versus the control; ^$$^ *p* < 0.05 and ^$^ *p* < 0.01 versus CP. PLE = *Piper longum* ethanolic extract: 200 mg/kg/day; PLE-H = *Piper longum* hexane fraction: 200 mg/kg/day; CP = cyclophosphamide: 5 mg/kg/day.

*Experimental design:* Extract and fractions of *Piper longum* were screened in a test battery consisting of the cytokinesis-block micronucleus assay and the chromosomal aberrations assay using OECD guidelines (OECD guidelines: Test no. 487: *in vitro* mammalian cell micronucleus test and test no. 473: *in vitro* mammalian chromosomal aberration test). The ethanolic extract and fractions (hexane, DCM, ethyl acetate, butanol, and aqueous) of *Piper longum* were tested in combination with cyclophosphamide (5 μg/mL) at the concentrations of 10, 20, and 40 μg/mL. The selected concentrations were found to be the least cytotoxic. Figure 10 shows the experimental design.

*Blood sample collection:* Human peripheral blood samples were used for CBMN and CA assays. Non-smoking, non-alcoholic, non-pregnant healthy adult volunteers (age between 18 and 25) were selected, and their blood samples were collected in a heparinized vacutainer and stored at 4°C. The study protocol was approved by Jamia Hamdard Institutional Ethics Committee (JHIEC-01/19), New Delhi, India.

*Analysis of cell viability by trypan blue exclusion:* Lymphocytes were incubated with the extract and fractions of *Piper longum* alone and in combination with cyclophosphamide (5 μg/mL) at various concentrations (Strober, 2015).

*CBMN assay and CA assay:* The culture was set up for 72 h; the extract and fractions were simultaneously incubated with cyclophosphamide for 24 h prior to harvesting. At 44 h, cytochalasin B (6 μg/mL) was exposed to arrest the cytokinesis process, while in the CA assay, Colcemid was added 90 min before the harvesting to arrest the cells at the metaphase. After incubation, cellular harvesting, hypotonic medium treatment, fixation, and Giemsa staining were performed.

*Microscopic evaluation*: A total of 4,000 binucleated cells for individual treatments were assessed using the standard measures for the identification of MNi (Fenech et al., 2003); while for the CA analysis, 300 well-spread metaphases were studied for each treatment (Ananthi et al., 2010).

### 3.6 Identification of markers by HPTLC

The setup comprised a CAMAG Linomat-V automatic sample applicator and a CAMAG TLC Scanner-3, both of which were equipped with WinCATS software (version 1.4.4). The pre-coated silica gel 60 F254 HPTLC plates served as the stationary phase. Linomat-V equipped with a 100-μL Hamilton syringe was used to apply samples to the stationary phase in 6-mm-broad bands. During the sample application, the nitrogen flow rate was 140 nL/s. A CAMAG twin trough glass chamber was saturated beforehand with the mobile phase (toluene:ethyl acetate:formic acid; 5:4:1, v/v/v) for 25 min, following a linear escalating manner of development located at a distance of 80 mm. Following this development, the plate was left to dry and was photographed in a CAMAG visualizing chamber using short and long UV wavelengths. Densitometric imaging was carried out at 325 nm in the CAMAG Scanner-3. The standard curve was prepared in accordance with the International Council for Harmonisation guidelines, and the validation parameters were addressed.

### 3.7 Metabolite fingerprinting of the *Piper longum* ethanol extract by UPLC-MS

The *Piper longum* ethanolic extract was subjected to UPLC-MS to detect the presence of various classes of phytoconstituents. UPLC was carried out using the Waters Acquity UPLC^(TM)^ system that was furnished with a binary solvent delivery system, an auto-sampler, a column controller, and an MS detector (serial no. JAA272; SYNAPT; Waters, Manchester, UK) connected and managed by MassLynx V 4.1. (Waters, United States). On a monolithic capillary silica-based C18 column (Acquity UPLC(R) BEH C18; 1.7 m; 2.1 × 100 mm), with the pre-column at an ambient temperature, chromatography was carried out using water (0.1% formic acid) (A) and acetonitrile (B) as the mobile phases. The separation was attained by the gradient mode (16 min, initially, 10% B; 0–5 min 40% B; 5–10 min 60% B; 10–13 min, 90% B; 13–15 min, 100% B; 15–16 min, 10% B). The mass and composition for precursor ions and fragment ions were calculated using MassLynx V 4.1 software (Zahiruddin et al., 2017).

### 3.8 Metabolite fingerprinting of the *Piper longum* hexane fraction by GC-MS

For hexane fraction analyses, GC-MS equipment with a CTC PAL auto-sampler with a mass spectrophotometer detector was used. The specimens were loaded into an Agilent HP-5MS column with a 5:1 split ratio (composed of 5% polymethylsiloxane; Agilent Technologies). The temperature of the inlet was fixed at 250°C. The temperature program for the oven was as follows: 50°C for 2 min, then 10°C/min to 200°C for 5 min, and then, 5°C/min to 250°C for 10 min. The carrier gas was helium, with a constant flow rate of 1.5 mL/min, with all ion source temperatures set to 250°C. The electron ionization energy was 70eV, and the complete mass scanning range was set at 50–700 amu. MSD ChemStation software was used to process data (Zahiruddin et al., 2020).

### 3.9 *In vivo* studies: Animals and ethics

Adult Wistar albino rats (both sexes), 8–10 weeks old, weighing 120–170 g, were acquired from the Central Animal House Facility of Jamia Hamdard, New Delhi. The experimental design was approved by the Institutional Animal Ethics Committee (IAEC) of Jamia Hamdard, New Delhi, [Committee for the Purpose of Control and Supervision of Experiments on Animals (CPCSEA registration number: 173/GO/Re/S/2000/CPCSEA)], India, under the protocol numbers 1665 and 1710. The 3R approach was used in the current study with the goal of reducing the number of animals using samples (serum and tissue) from negative controls (vehicle treated animals) and positive controls (cyclophosphamide treated animals) under the aforementioned protocol number. The samples were analyzed in comparison with the test agents. All studies involving animals were reported in accordance with the Animal Research: Reporting of *In Vivo* Experiments (ARRIVE) guidelines 2.0 for reporting experiments involving animals. In polypropylene cages with a 12:12 h light-dark cycles, the animals were housed in environments with controlled humidity and temperature (25°C ± 2°C; 55–65%). Pellet feed was given to the animals, and they had unrestricted access to food and water. The experimental design to conduct acute oral toxicity (fixed-dose procedure), sub-acute toxicity (repeated for 28 days for oral toxicity), and the genomic instability study were adopted from the OECD guidelines for the testing of chemicals 423, 407, and 474, respectively.

#### 3.9.1 Systemic toxicity studies: Acute and sub-acute oral toxicity studies

In the acute toxicity study, the *Piper longum* ethanolic extract and hexane fraction were administered as oral suspensions of 2,000 mg/kg to three animals of each gender (*n* = 6) and control animals (*n* = 6) with 0.5% CMC. Fourteen days’ post-treatment observations were made to track physical alterations, especially for the first 4 h of drug administration.

In the sub-acute toxicity study, rats (*n* = 10, five males and five females) were orally administered with three graded doses (200, 400, and 800 mg/kg/day) of the *Piper longum* ethanolic extract and the *Piper longum* hexane fraction, while the control group received vehicle for 28 days. Additionally, a satellite group of 10 animals in the control and in the high-dose group (800 mg/kg/day) for the observation of reversibility, persistence, or delayed occurrence of toxic effects, for 14 days post-treatment, was also included in the study.

After the last day of the drug treatment, the animals were anesthetized for blood sample collection and euthanized to collect the vital organs. The animals were anaesthetized using ketamine and xylazine (80 mg/kg and 8 mg/kg, respectively; injected intraperitoneally) and sacrificed in a CO_2_ inhalation chamber. The semi-automated biochemistry analyzer “Diasys Photometer5010” was used to evaluate the samples in accordance with the manufacturer’s instructions for the kits obtained from DiaSys Diagnostics, Private Limited, India.

#### 3.9.2 Effects of *Piper longum* on the cyclophosphamide-induced model of genomic instability

##### 3.9.2.1 Experimental design

Wistar albino rats (*n* = 10, five males and five females) were divided into the following groups: 1) vehicle treatment (negative control), 2) cyclophosphamide (CP: positive control)-exposed group: 5 mg/kg, 3) *Piper longum* ethanolic extract: 200 mg/kg, 4) co-treatment with cyclophosphamide: 5 mg/kg + the *Piper longum* ethanolic extract: 200 mg/kg, 5) *Piper longum* hexane fraction: 200 mg/kg, and 6) co-treatment with cyclophosphamide: 5 mg/kg + the *Piper longum* hexane fraction: 200 mg/kg.

Drugs were subsequently administered by oral gavages for 28 days, and OECD guideline 474 was used for sample size prediction. The dose for evaluating the antigenotoxic potential of the *Piper longum* ethanolic extract and the *Piper longum* hexane fraction was chosen as 200 mg/kg/per day, as it showed no toxic signs, while for cyclophosphamide, the human dose was converted into a rat dose (Nair and Jacob, 2016).

##### 3.9.2.2 *In vivo* bone marrow micronuclei assays

The method of Schmid W (1976)’s study was adopted to acquire micronucleated polychromatic erythrocytes (MNPCEs). Each animal was assessed for 1,000 polychromatic erythrocytes (PCEs) to estimate MNPCE frequency. The PCE:NCE ratio was measured by scoring 1,000 erythrocytes per animal to assess cytotoxicity (Waters et al., 1990).

##### 3.9.2.3 Diphenylamine assay

DNA fragmentation in liver and brain homogenates was determined using diphenylamine-based colorimetric assays (Gibb and Gercel-Taylor, 2000; Gercel-Taylor, 2005).

##### 3.9.2.4 Analyses of reactive oxygen species, MDA, and glutathione estimation

ROS generation in liver and brain (hippocampus) tissue was quantified by the dye 2′,7′-dichlorofluorescin diacetate (DCFH-DA) (Liu et al., 2018). Total protein was estimated by Lowry’s colorimetric procedure (Lowry et al., 1951). MDA estimation was adopted from the method in a study by Ohkawa et al. (1979), while glutathione assessment was adopted from Hissin and Hilf (1976)

##### 3.9.2.5 Evaluation of 8-hydroxyguanosine

Hippocampus and hepatic tissue was analyzed by using GENLISA™ ELISA kit, Krishgen Biosystems, India. The producer’s directions were strictly followed while performing the experiment.

##### 3.9.2.6 Histology

H&E-stained brain (hippocampus) and liver tissue sections were examined under a microscope. The liver slice was ranked by a severity score to obtain the total histological index (Supplementary Table S10). The hippocampus slice was observed to conclude the percent pyknosis (Iqbal et al., 2019).

##### 3.9.2.7 Expression of γH2AX

The liver and hippocampus tissue samples were embedded in paraffin after being fixed in 10% neutral-buffered formalin for 24 h. For immunohistochemistry (IHC) staining, a standard approach was used (Crosby et al., 2014). By calculating the average proportion of γH2AX-positive cells per 1,000 cells, the immuno-expression was assessed.

### 3.10 Statistical analyses

One-way analysis of variance (ANOVA) was used to analyze the data, and then, Tukey–Kramer multiple-comparison tests were performed. Fisher’s exact test was used to determine the statistical significance of chromosomal abnormalities and the frequency of micronucleated binuclear cells, whereas the chi-square test was used to determine the frequency of micronucleated erythrocytes in mammals. Prism software program was used to conduct all statistical analyses (version 8, GraphPad, San Diego, CA). Significance was determined as *p* < 0.05.

## 4 Discussion

**FIGURE 11:**
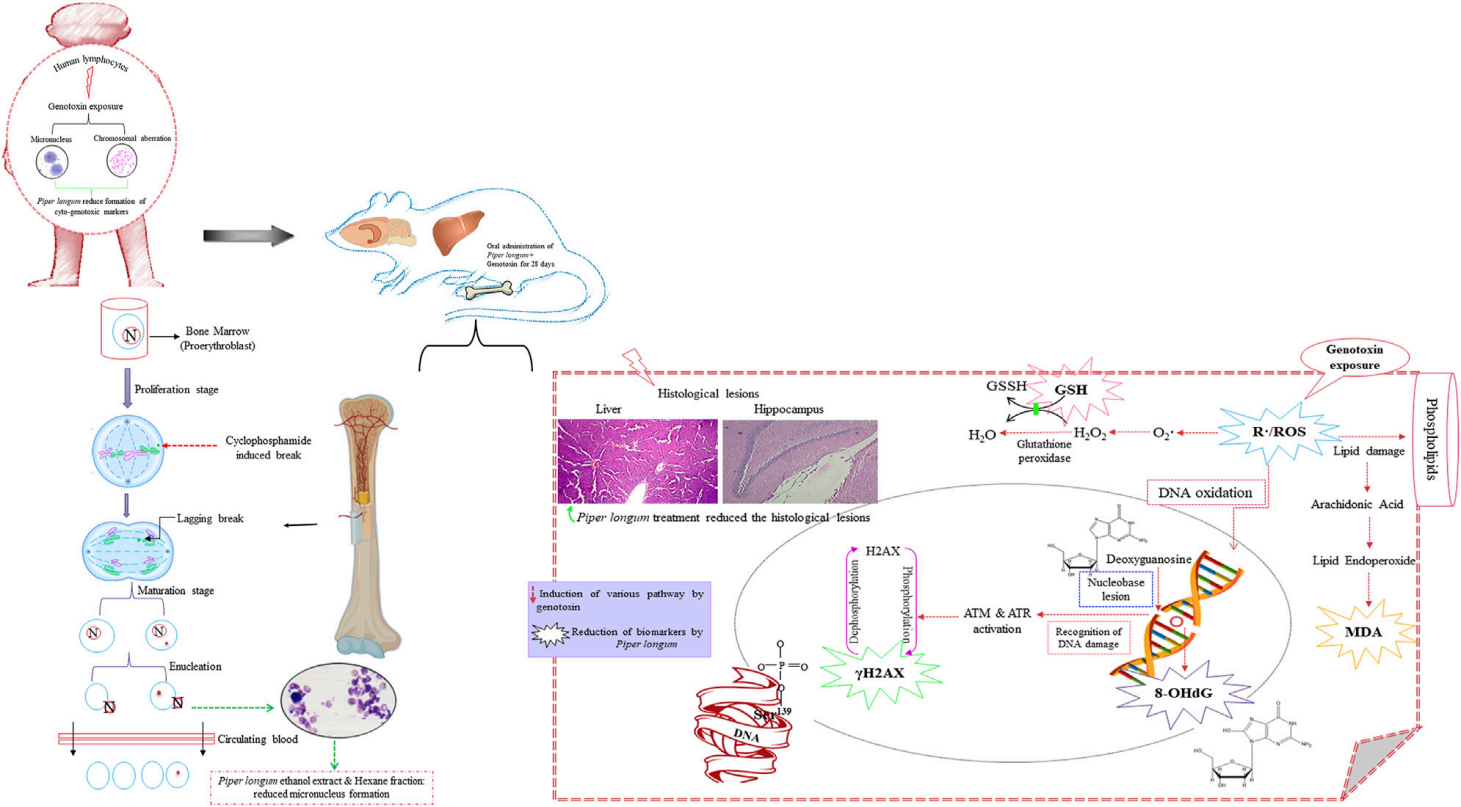
Graphic illustration of the effect of *Piper longum* on rats exposed to cyclophosphamide.

The current investigation reports the genome-stabilizing action of *Piper longum* on cyclophosphamide-induced genotoxicity using *in vitro* and *in vivo* models. Both the *Piper longum* ethanolic extract and hexane fraction demonstrated significant reduction in cytogenetic markers (micronucleus and chromosomal aberrations) against cyclophosphamide-induced damage in human peripheral lymphocytes. Furthermore, their treatment maintained the genomic stability by reducing oxidative DNA damage and γH2AX, a DNA double-strand break (DSB) marker in tissues, and preserving vital tissues against histopathological lesions induced by the chronic exposure of cyclophosphamide (Figure 11). Under *in vivo* conditions, *Piper longum* did not cause any noticeable systemic toxicity, as determined in acute and sub-acute toxicity studies conducted in accordance with OECD guidelines.

The effect of the crude ethanolic extract of *Piper longum* was studied in an acellular medium using plasmid pBluescript SK(-) DNA against H_2_O_2_-induced strand breaks. The experiment showed three confirmations in untreated DNA samples: supercoiled conformation, open circles resulting from single-strand breaks, and linear confirmation resulting from double-strand breaks, which are in agreement with results from earlier reports (Russo et al., 2006; Horvathova et al., 2014). The H_2_O_2_ treatment resulted in the cleavage of supercoiled DNA, while the co-incubation of the *Piper longum* ethanolic extract with H_2_O_2_ caused retention in the supercoiled DNA confirmation. This test provided preliminary evidence for the effectiveness of the extract, following which the bioactive moieties in the crude extract were segregated by fractionation and further validated for genoprotection.

Cytogenetic hallmarks in lymphocytes such as chromosomal aberrations and micronuclei are reliable markers for establishing cellular and nuclear dysfunction caused by genotoxin exposure (Hayashi, 2016). Thus, the effects of the *Piper longum* ethanol extract along with fractions (hexane, dichloromethane, ethyl acetate, butanol, and aqueous fraction) were screened for the frequency of these markers in peripheral blood lymphocytes induced by cyclophosphamide. Our results depicted an escalation in the formation of these markers by cyclophosphamide treatment, which is coherent with reports suggesting the clastogenic nature of cyclophosphamide on various cell lines such as Hep G2 cells and lymphocytes, using the micronucleus and chromosomal aberrations as markers (McCarroll et al., 2008). Furthermore, simultaneous incubation experiments with the *Piper longum* ethanolic extract and cyclophosphamide significantly reduced MNi and CA, which are indicative of genoprotection. Also, amid various fractions, only the hexane fraction was found to produce a significant reversal in these biomarkers.

Next, the *Piper longum* extract and fraction were examined in a cyclophosphamide-induced rodent model of genomic instability to validate *in vitro* findings and to better understand molecular mechanisms involved in genoprotection. We used cyclophosphamide as a positive control to induce genomic instability, which is in accordance with OECD guideline no. 474. Cyclophosphamide is an antineoplastic medicine that has been shown to be effective against a wide range of hematological cancers and autoimmune diseases. The clinical utilization of cyclophosphamide is often limited because of its adverse reactions, which include nausea, vomiting, alopecia, bone marrow suppression, hepatotoxicity, nephrotoxicity, cardiotoxicity, immunotoxicity, mutagenicity, teratogenicity, and carcinogenicity (LH et al., 1991). Patients receiving cyclophosphamide chemotherapy have demonstrated a high incidence of cytogenetic markers (such as micronucleus formation, nuclear abnormalities, chromosomal aberrations, and sister chromatid exchanges) and induced gene expression profiles related to oxidative damage, cell cycle control, and apoptosis in lymphocytes (Goldberg et al., 1990; McDiarmid et al., 1990; Zúñiga et al., 1996; Sakamoto-Hojo et al., 2003), indicating genotoxicity by cyclophosphamide exposure. Aside from that, there have been reports of cancer among healthcare personnel as a result of occupational exposure to cyclophosphamide (Sottani et al., 2010). Thus, cyclophosphamide generates a clinically relevant pathophysiology to assess the genoprotection of novel therapeutics. Furthermore, compounds that avert genotoxic events impacting the DNA can act by different mechanisms. They might directly interact with toxins or block their impact through metabolic activation or the stimulation of their detoxifying enzymes, which can be evaluated in a pre-treatment or co-treatment study regimen. While other agents might act after the damage, by endorsing DNA repair, which can be detected in post-treatment, increasing the fidelity of DNA replication, inhibiting error prone replication, or suppressing the growth and replication of cells with damaged DNA. Hence, it is possible to recommend mechanisms of action for a test drug based on the treatment regimen.

The mammalian erythrocyte micronucleus test is another cytogenetic assay, using erythrocytes derived from the bone marrow for identifying injury to chromosomes or the mitotic apparatus of cells (Jain and Pandey, 2019). The frequency of the micronucleus in polychromatic erythrocytes was increased upon 28 days of oral exposure to cyclophosphamide at the dose of 5 mg/kg/day, while simultaneous administration of the *Piper longum* extract and its fraction with cyclophosphamide reversed the micronucleus formation in polychromatic erythrocytes of the bone marrow. The findings confirm the cyto(geno)toxicity of cyclophosphamide, which is consistent with earlier reports, wherein cyclophosphamide causes an increased incidence of MNi and CA in rat bone marrow cells (Hosseinimehr and Karami, 2005; Ahmadi et al., 2008). The liver and brain appear to be significant targets for cyclophosphamide toxicity (Akomolafe et al., 2020); thus, it was critical to examine the hepato-genotoxicity and neuro-genotoxicity. We examined genomic DNA damage in tissues, measured as the percentage of DNA fragmentation, by the diphenylamine colorimetry test. The present experiment demonstrated that cyclophosphamide exposure resulted in raised genome fragmentation in tissues, while the co-exposure of the extract and fraction with cyclophosphamide reduced the percentage of DNA fragmentation. These results confirm that the *Piper longum* extract and fraction efficiently offered genome protection in the liver and hippocampus against the chronic exposure of genotoxins.

Oxidative stress is a critical signaling pathway by which DNA damage occurs and is also known to inhibit DNA repair mechanism (Gonzalez-Hunt et al., 2018). It is suggested that the cellular mechanism of toxicities caused by cyclophosphamide is related to the stimulation of oxidative stress through the production of free radicals in healthy tissues (Basu et al., 2015; Bhattacharya et al., 2022). The present examination showed higher generation of ROS and MDA, with depleted GSH levels by cyclophosphamide intoxication in both the liver and the hippocampus. The *Piper longum* extract and hexane fraction co-administration with cyclophosphamide mitigated ROS levels, lipid peroxidation (MDA levels), and restored GSH levels in control rats. The observations are consistent with those of earlier reports (Wakade et al., 2008; Thomas et al., 2009), where the *Piper longum* extract generated defense against oxidative stress induced by monosodium glutamate and Adriamycin. This modulatory effect is due to the ability of *Piper longum*, which can serve as an antioxidant by removing free radicals, diminishing pro-oxidative burden, and retaining antioxidant enzymes. Our findings evidently indicate that the *Piper longum* extract and hexane fraction generate efficient safeguards in contradiction to the genotoxin-exposed redox imbalance in vital tissues.

The interaction of reactive oxygen species with genomic material leads to the generation of oxidized DNA bases and DNA strand breaks. Specifically, 8-OHdG is an abundantly generated oxidative DNA abrasion. Thus, we evaluated the levels of 8-OHdG, which determine the magnitude and pathobiological relevance of oxidative genomic damage (Farmer, 2004). In the current study, chronic exposure to cyclophosphamide increased the production of the oxidative genomic damage product, as presented by the rise of 8-OHdG, whereas the co-treatment of cyclophosphamide with the *Piper longum* ethanol extract resulted in a more efficient reduction of 8-OHdG in the liver and hippocampus tissue in both genders, in comparison to the hexane fraction treatment. This effect of *Piper longum* can be credited due to the recovery from cyclophosphamide-associated reactive oxygen species formation, consequently mitigating the hydroxyl radical formation and, therefore, providing a shield to DNA against purine injury. The maintenance of the balance between the formation of free radicals and increased antioxidant enzyme activity may be the cause for the genome-stabilizing effect. Numerous research teams have looked into the positive relationship between antioxidants and genomic integrity (Cooke et al., 2002; Kaur et al., 2019; Bhattacharya et al., 2022). Furthermore, the phytochemical composition is accountable for beneficial therapeutic activity of herbs (Forni et al., 2019). We identified the presence of alkaloids, mainly piperine and piperlongumine, lignans, terpenoids, fatty acids, and flavonoids in the *Piper longum* extract and fraction using modern chromatographic and mass spectroscopic techniques. Reports have suggested that these phytochemicals are often associated with the genoprotective action against various types of physical, chemical, and biological genotoxin exposure, as demonstrated under cell cultures and preclinical scenarios (Aprotosoaie et al., 2019; Shruthi and Shenoy, 2022). Furthermore, in addition to its anti-oxidative potential, *Piper longum* might have exhibited protective effects on the liver and hippocampus by altering the inflammatory cascade, endogenous liver metabolizing enzymes such cytochrome P450, notably CYP3A4, and as yet unidentified mechanisms.

Liver histology was examined in the current experimental setup, wherein cyclophosphamide intoxication caused extreme lesions, as stated in the aforementioned results. These injuries provide insight into microarchitectural details of the liver, for instance, steatosis is a feature of fatty liver/accumulation of triacylglycerol into hepatocytes (Nassir et al., 2015); apoptosis is characterized by nuclear condensation (Krishna, 2017); inflammation is often associated with fibrosis (Park et al., 2014); congestion/dilation of the central vein and sinusoidal dilation are consequences of circulatory impairments (Hilscher and Sanchez, 2016), and hyperplasia of the bile duct is associated with toxic inflammatory insult (Horvathova et al., 2014). The examined tissue injuries are consistent with earlier reports, whereby chemotherapy drugs such as cyclophosphamide caused identical abnormalities in a preclinical setting and in chemotherapy recipients (Robinson, 2009; Ince et al., 2014; Sharma et al., 2014; Abdelfattah-Hassan et al., 2019; Fateh et al., 2019), suggesting a clinical simulation of the experimental design. Additionally, the *Piper longum* ethanol extract when co-administered with cyclophosphamide reversed the histological lesion more efficiently than the hexane fraction. Earlier reports (Christina et al., 2006) are in line with our findings, where the liver histopathological lesion was reduced by the *Piper longum* ethanolic extract, administered for 28 days for CCl4-induced liver injury in rats, and they confirmed the hepatoprotective and antifibrotic nature of *Piper longum*.

We further evaluated the microstructure of the hippocampus for neuronal damage, as cyclophosphamide is extremely poisonous to neurons; it breaches the brain barrier and is associated with memory impairment (Akomolafe et al., 2020). In our experiment, cyclophosphamide exposure caused an increment in the percentage of pyknosis, which was more severe in the CA1 region than in DG. This outcome is associated with the neuroarchitectural differences in the hippocampus: DG is relatively invulnerable to insults, whereas CA1 is susceptible to several toxins (Alkadhi, 2019). According to recent research, cyclophosphamide suppresses neurogenesis by first causing intracellular oxidative damage and then disrupting the blood–brain barrier and opening a doorway for potentially neurotoxic chemicals to enter the cerebellum (Yang et al., 2010). “Chemo brain” is the term used to describe the neurotoxic effects of chemotherapy (O’Farrell et al., 2013). Furthermore, the co-treatment of the *Piper longum* ethanol extract with cyclophosphamide presented higher neuronal protection in the CA1 region than in the hexane fraction, whereas in the DG region, both the extract and fraction show an almost equal neuronal protection, as measured in terms of the percentage of pyknosis. Similar reports have been documented before, where the administration of the *Piper longum* ethanol extract in rats prevented neuronal injury induced by intranigral injections of lipopolysaccharide (He et al., 2016) and a glioma rodent model (Subramanian et al., 2010). These studies attributed the antioxidant and anti-inflammatory property of *Piper longum* for neuronal protection.

Amid various forms of damage that are inflicted by genotoxin exposure, the most severe is the DNA DSB, which damages the genome’s structural stability and is a serious lesion that, if left untreated, can result in genomic instability, cancer, mutations, or cell death (Iliakis et al., 2019). γH2AX is a primary predictor of DNA double-strand breaks and essential in the regulation of the DNA damage response, which is demonstrated by *in vitro*, *in* vivo, and clinical experiments (Bourton et al., 2011; Kopp et al., 2019). Members of the phosphatidyl-inositol-3-kinase-related kinases (PIKKs) family, phosphorylate H2AX histones near the DNA break, shortly after the DSB is generated. This phosphorylated version of histone H2AX, known as γH2AX, is a genotoxicity biomarker (Collins et al., 2020). Our experiment presented a rise in γH2AX expression in hepatocytes and the hippocampus after repeated administration of cyclophosphamide. Cyclophosphamide leads to cross-linking in DNA strands and induces γH2AX expression in oocytes and in cultured mouse ovarian granulosa cells (Bourton et al., 2011; Petrillo et al., 2011). Our findings further found that the co-treatment of cyclophosphamide with the *Piper longum* ethanol extract demonstrated a more efficient reduction of γH2AX foci in the hippocampus than in the hexane fraction, whereas in liver tissue, the extract and fraction showed a nearly equal reduction of γH2AX immunopositivity. Numerous studies have demonstrated that γH2AX immune-suppression is consequently related to guarding the genome against DNA DSBs (Chethna et al., 2018; Turrini et al., 2018; Deng et al., 2020). Thus, the outcome of *Piper longum* may have resulted from the decreased DNA double-strand break generation.

Furthermore, systemic toxicological studies in rats show a strong association across preclinical and clinical contexts of safety. Moreover, to strengthen the assurance in medicinal plant security, evidence for toxicity studies should be gathered (Li et al., 2019). Previously, only one report on the toxicity analysis of the *Piper longum* fruit was available, conducted on mice using a single dose (Shah et al., 1998), which was not in accordance with OECD guidelines. Also, piperine, one of the major bioactive moieties in *Piper longum*, is linked with elevated aspartate aminotransferase and alkaline phosphatase levels, indicating substantial hepatic damage in murines (Haq et al., 2021). Thus, it was essential to conduct acute and sub-acute toxicity studies following the international guidelines. During 28 days, the repeated administration of the *Piper longum* extract and hexane fraction at three graded dosages (200, 400, and 800 mg/kg) was carried out; only the hexane fraction at dose of 800 mg/kg showed a substantial increase in ALP, AST, and ALT, indicative of hepatic insults. However, other biochemistry parameters (such as cholesterol, HDL, VLDL, triglycerides, hematological indices, bilirubin, urea, uric acid, creatinine, protein, calcium, and glucose levels) were not altered by the extract and fraction treatment. Thus, our findings indicate that the *Piper longum* extract and fraction did not show any signs of hepato-, nephro-, or hematotoxicity, as presented in biochemical data, supporting the safety of *Piper longum* for use in food applications. Moreover, further investigation will be required to comprehend the action of *Piper longum* in a tumor-bearing animal model to ascertain mechanisms of cyclophosphamide in inducing DNA damage in cancer cells; exploring the post-treatment response of *Piper longum* after cyclophosphamide intoxication, where it might exhibit an antagonistic effect in normal and cancer cells and assess the selectively of *Piper longum* toward normal cells. Also, for the efficient translation of *Piper longum* in a clinical scenario for chemotherapy-receiving patients, it will be interesting to examine the response in different cell types and against various chemotherapeutics such as 5-fluorouracil, bleomycin, and methotrexate.

## 5 Conclusion

We report the genoprotective action of the *Piper longum* extract, for the first time, as confirmed in an acellular medium, human peripheral blood lymphocytes, and cyclophosphamide-induced rodent models of genomic instability. Multiple biomarkers such as micronucleus formations, chromosomal aberrations, oxidative stress markers, histopathological lesions, 8-OHdG, and γH2AX were examined in the genotoxin-induced rodent model. *Piper longum* treatment reduced the genomic lesion formation and diminished the DNA damage response pathway in rats. Apart from shifting the cellular and molecular signaling markers toward genomic stability, *Piper longum* treatment also conserved the microarchitectural details of the liver and hippocampus, which deteriorated as a secondary pathological consequence of genotoxin exposure. Upcoming investigations may examine the potential role played by *Piper longum* therapy in DNA repair mechanisms.

## Data Availability

The original contributions presented in the study are included in the article/Supplementary Material; further inquiries can be directed to the corresponding author.

## Glossary

8-OHdG: 8-Hydroxy-2-deoxyguanosine
ARRIVE: Animal Research Reporting of *In Vivo* Experiments
CA: Chromosomal aberrations
CA1: Cornu ammonis 1
CBMN: Cytokinesis-block micronucleus test
CPCSEA: Committee for the Purpose of Control and Supervision of Experiments on Animals
CP: Cyclophosphamide
DCFH-DA: 2′,7′-dichlorofluorescin diacetate
DCM: Dichloromethane
DG: Dentate gyrus
DNA: Deoxyribonucleic acid
DPA: Diphenylamine
DSB: DNA double-strand break
**GC**-**MS**: Gas chromatography–mass spectrometry
GSH: Glutathione
H&E: Hematoxylin and eosin
**H**_**2**_ **O**_**2**_: Hydrogen peroxide
HPTLC: High-performance thin-layer chromatography
IAEC: Institutional Animal Ethics Committee
IHC: Immunohistochemistry
JHIEC: Jamia Hamdard Institutional Ethics Committee
MDA: Malondialdehyde
MNPCE: Micronucleated polychromatic erythrocyte
OECD: Organisation for Economic Co-operation and Development
PIKK: Phosphatidylinositol-3-kinase-related kinase
PLE: *Piper longum* ethanolic extract
PLE-H: Hexane fraction of *Piper longum*
ROS: Reactive oxygen species
Rt: Retention time
TLC: Thin-layer chromatography
**UPLC**-**MS**: Ultra-high performance liquid chromatography–mass spectroscopy
