# Supplementary material for: Amelioration of cyclophosphamide-induced DNA damage, oxidative stress, and hepato- and neurotoxicity by Piper longum extract in rats: The role of γH2AX and 8-OHdG
Source: Front Pharmacol. 2023 Mar 10;14:1147823. doi: 10.3389/fphar.2023.1147823 (PMC10036401; doi:10.3389/fphar.2023.1147823)
Supplement: Supplementary file 1 [file DataSheet1.docx]

Supplementary Material

Amelioration of cyclophosphamide-induced DNA damage, oxidative stress, hepato- and neurotoxicity by *Piper longum* extract in rats: Role of γH2AX and 8-OHdG

Vaishali Yadav, Anuja Krishnan, Sultan Zahiruddin, Sayeed Ahmad, Divya Vohora^*^

*** Correspondence:** Prof. Divya Vohora, Email: [dvohra@jamiahamdard.ac.in](mailto:dvohra@jamiahamdard.ac.in)

**Supplementary Figure 1S**

**A**


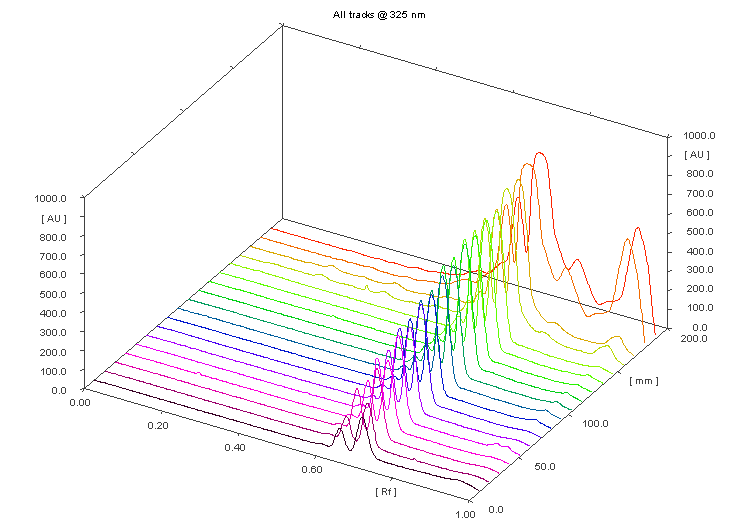


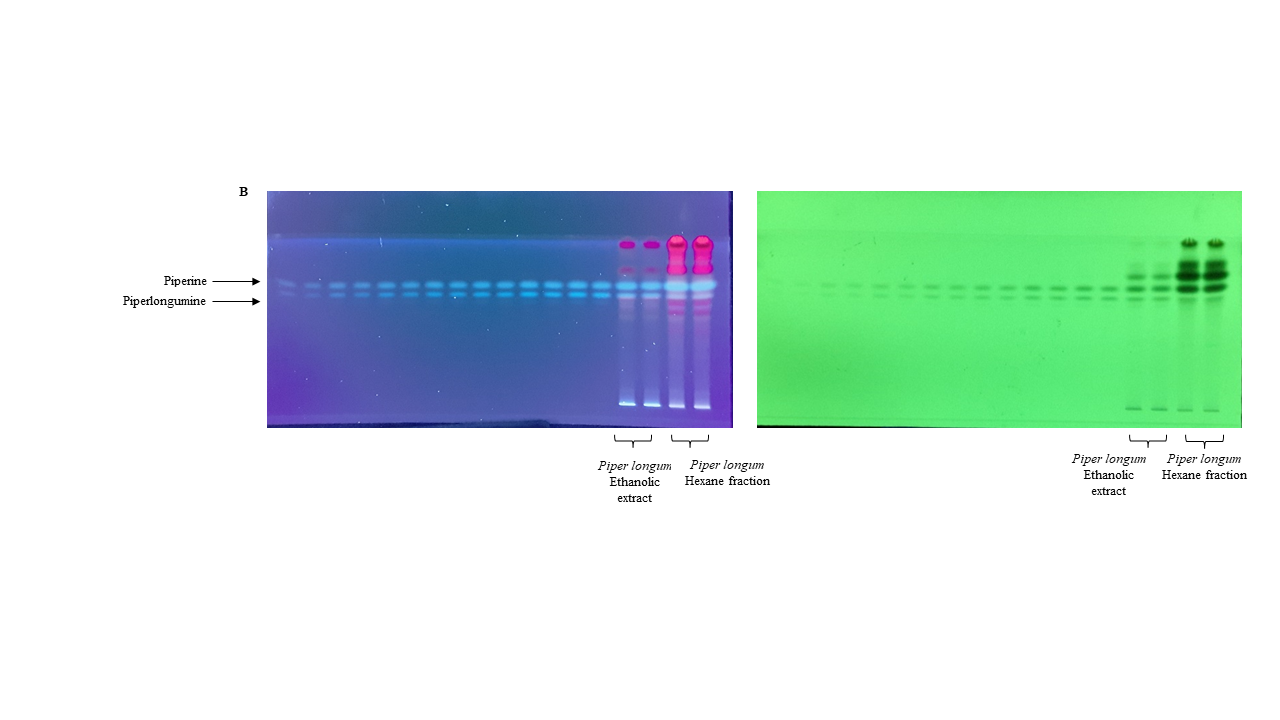


**C D E**


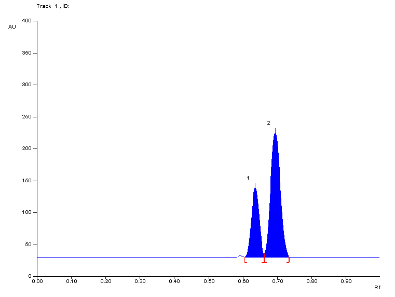

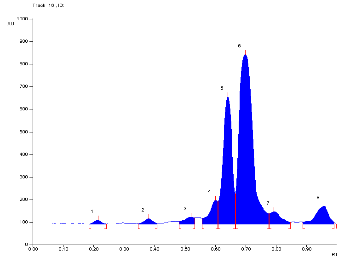

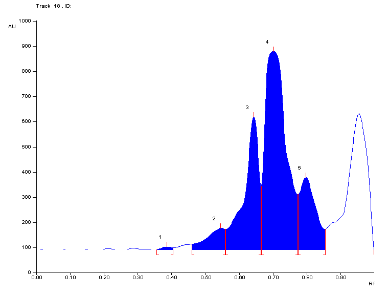


**Table 1S**: Method validation parameters for piperine and piperlongumine in *Piper longum* extract and hexane fraction proposed by HPTLC method

| S. No | Parameters | Ethanolic extract of *Piper longum* | | Hexane fraction of *Piper longum* | |
| --- | --- | --- | --- | --- | --- |
|  |  | Piperine | Piperlongumine | Piperine | Piperlongumine |
| 1 | Linearity range (ng/spot) | 100-1000 | 100-1000 | 100-1000 | 100-1000 |
| 2 | Correlation coefficient (r^2^) | 0.9983 | 0.9992 | 0.9976 | 0.9988 |
| 3 | Regression equation | Y= 14.907x+5304 | Y=10.946x+1801.4 | Y= 14.805x+5378.3 | Y= 11.117x+1778.6 |
| 4 | Standard error of intercept | 152.79 | 74.11 | 178.78 | 95.54 |
| 5 | Limit of detection (LOD) (ng) [3*SD/S] | 33.8 | 22.33 | 39.84 | 28.36 |
| 6 | Limit of quantification (LOQ) (ng) [10*SD/S] | 102.5 | 67.7 | 120.75 | 85.94 |
| 7 | Instrumental precision (RSD, n=6) | 0.82% | 0.98% | 0.74% | 0.86% |
| 8 | Percentage content | 0.91% | 0.66% | 2.91% | 1.83% |

**Supplementary Figure 2S**


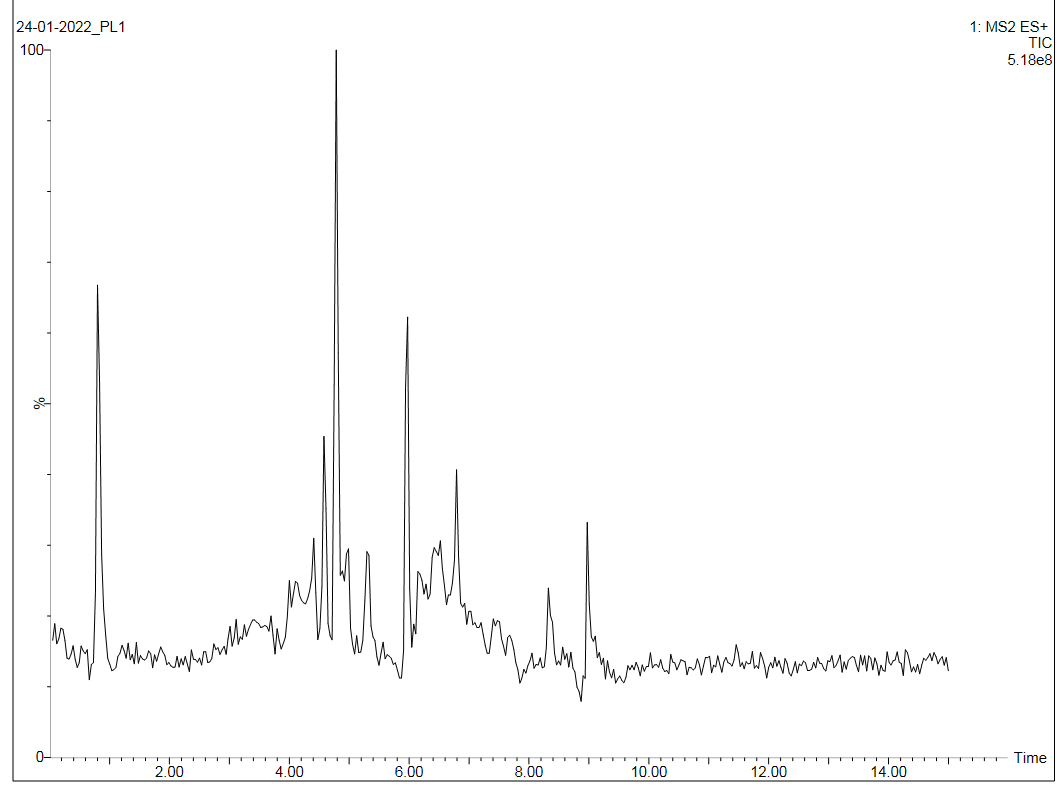


**Table S2**: Qualitative analysis of phytoconstituents in ethanol extract of *Piper longum* by UPLC-MS

| **S. No.** | **Rt** | **Compound** | **Class** | **m/z**  **Experimental**  **[M+H]^+^** | **Reference**  **(Mass ID)** | **Molecular**  **formula** | **m/z Theoretical** |
| --- | --- | --- | --- | --- | --- | --- | --- |
| 1 | 4.408 | Aristolactam BII | Alkaloid | 278.90 | 162739 | C17H13NO3 | 280.29 |
| 2 | 4.408 | Piperlongumine | Alkaloid | 318.15 | 637858 | C17H19NO5 | 318.13 |
| 3 | 4.408 | Rosmarinic acid | Phenolics | 343.17 | 5281792 | C18H16O8 | 343.08 |
| 4 | 4.408 | Pipernonaline | Alkaloid | 343.30 | 9974595 | C21H27NO3 | 342.4 |
| 5 | 4.579 | Apigenin | Flavonoid | 271.96 | 5280443 | C15H10O5 | 271.06 |
| 6 | 4.579 | Naringenin | Flavonoid | 273.98 | 11471237 | C15H12O5 | 273.07 |
| 7 | 4.579 | Piperlonguminine | Alkaloid | 274.23 | 5320621 | C16H19NO3 | 274.14 |
| 8 | 4.579 | 4,5-Dihydropiperlonguminine | Alkaloid | 276.30 | 12682184 | C16H21NO3 | 276.34 |
| 9 | 4.783 | Piperine | Alkaloid | 286.08 | 638024 | C17H19NO3 | 286.14 |
| 10 | 4.783 | Isopiperine | Alkaloid | 286.21 | 1548913 | C17H19NO3 | 286.34 |
| 11 | 4.783 | Kaempferol | Flavonoid | 287.15 | 5280863 | C15H10O6 | 287.05 |
| 12 | 4.783 | Piperanine | Alkaloid | 288.03 | 5320618 | C17H21NO3 | 288.15 |
| 13 | 5.97 | Pellitorine | Alkaloid | 224.20 | 5318516 | C14H25NO | 224.201 |
| 14 | 6.51 | Achilleamide | Alkaloid | 236.1113 | 11118018 | C15H25NO | 235.36 |
| 15 | 6.51 | Retrofractamide B | Alkaloid | 356.15 | 5372162 | C22H29NO3 | 356.5 |
| 16 | 6.51 | Sylvatine | Lignan | 384.32 | 90472536 | C24H33NO3 | 383.5 |
| 17 | 6.51 | Guineensine | Alkaloid | 385.26 | 6442405 | C24H33NO3 | 384.253 |
| 18 | 6.79 | Piperolactam A | Alkaloid | 266.42 | 3081016 | C16H11NO3 | 266.081 |
| 19 | 7.47 | 2,4-Hexadecadienamide, N-(2-methylpropyl)-, (E,E)- | Alkaloid | 308.26 | 6442402 | C20H37NO | 308.5 |
| 20 | 7.47 | Piperolein B | Alkaloid | 344.24 | 21580213 | C21H29NO3 | 344.5 |
| 21 | 8.32 | Pipericine | Alkaloid | 336.30 | 9974234 | C22H41NO | 336.6 |

**Supplementary Figure 3S**

**Table S3**: Phytochemical analysis and identification of major compounds in hexane fraction of *Piper longum* by GCMS

| **S. No.** | **Rt** | **Compound** | **Class** | **Reference**  **(Mass ID)** | **Molecular**  **formula** |
| --- | --- | --- | --- | --- | --- |
| 1 | 6.52 | Tetradecene | Straight chain alkene | 14260 | C14H28 |
| 2 | 7.53 | Limonene | Terpenoids | 22311 | C10H16 |
| 3 | 8.90 | Palmitelaidic acid | Unsaturated fatty acid | 5282745 | C16H30O2 |
| 4 | 8.94 | Linalool | Terpenoids | 6549 | C10H18O |
| 5 | 9.76 | Cetyl alcohol | Fatty alcohol | 2682 | C16H34O |
| 6 | 9.97 | Butanoic acid | Saturated fatty acid | 264 | C4H8O2 |
| 7 | 10.18 | Docosanoic acid | Saturated fatty acid | 8215 | C22H44O2 |
| 8 | 10.22 | Heneicosanoic acid | Saturated fatty acid | 16898 | C21H42O2 |
| 9 | 10.35 | Octadecanoic acid | Saturated fatty acid | 5281 | C18H36O2 |
| 10 | 10.54 | 1-Tridecanol | Long-chain fatty alcohol | 8207 | C13H28O |
| 11 | 10.73 | Octanoic acid | Saturated fatty acid | 379 | C8H16O2 |
| 12 | 10.73 | Capric Acid | Saturated fatty acid | 2969 | C10H20O2 |
| 13 | 10.73 | Lauric acid | Saturated fatty acid | 3893 | C12H24O2 |
| 14 | 11.30 | Eicosane | Straight chain alkane | 8222 | C20H42 |
| 15 | 11.52 | Nonadecane | Straight chain alkane | 12401 | C19H40 |
| 16 | 11.52 | Pentacosane | Straight chain alkane | 12406 | C25H52 |
| 17 | 11.52 | Tetradecane | Straight chain alkane | 12389 | C14H30 |
| 18 | 11.58 | Citral | Terpenoids | 638011 | C10H16O |
| 19 | 11.66 | Triacontane | Straight-chain alkane | 12535 | C30H62 |
| 20 | 11.84 | Tridecanol | Fatty alcohol | 8207 | C13H28O |
| 21 | 11.94 | 7-Hexadecenal | Fatty aldehyde | 5364438 | C16H30O |
| 22 | 12.04 | Cetyl alcohol | Fatty alcohol | 2682 | C16H34O |
| 23 | 12.04 | 1-Heptadecanol | Fatty alcohol | 15076 | C17H36O |
| 24 | 12.19 | Octadecane | Straight-chain alkane | 11635 | C18H38 |
| 25 | 12.32 | 2,4-Decadienal | Unsaturated fatty aldehyde | 5283349 | C10H16O |
| 26 | 12.46 | Tetradecane | Straight chain alkane | 12389 | C14H30 |
| 27 | 13.22 | Hentriacontane | Long-chain alkane | 12410 | C31H64 |
| 28 | 13.34 | Pentatriacontane | Long-chain alkane | 12413 | C35H72 |
| 20 | 13.49 | Nonadecane | Straight chain alkane | 12401 | C19H40 |
| 30 | 13.49 | Pentacosane | Straight chain alkane | 12406 | C25H52 |
| 31 | 13.84 | Capraldehyde | Saturated fatty aldehyde | 8175 | C10H20O |
| 32 | 13.90 | Heptadecane | Straight chain alkane | 12398 | C17H36 |
| 33 | 13.90 | Pentadecane | Straight chain alkane | 12391 | C15H32 |
| 34 | 14.45 | Tricosane | Straight chain alkane | 12534 | C23H48 |
| 35 | 14.57 | Eicosane | Straight chain alkane | 8222 | C20H42 |
| 36 | 14.92 | Squalane | Hydrocarbon | 8089 | C30H62 |
| 37 | 15.01 | n-Docosane | Straight chain alkane | 12405 | C22H46 |
| 38 | 15.49 | Nerolidol | Terpenoids | 5284507 | C15H26O |
| 39 | 15.49 | Pinene | Monoterpene | 6654 | C10H16 |
| 40 | 15.73 | Tritriacontane | Straight chain alkane | 12411 | C33H68 |
| 41 | 15.84 | Tetratetracontane | Straight chain alkane | 23494 | C44H90 |
| 42 | 16.14 | Docosane | Straight chain alkane | 12405 | C22H46 |
| 43 | 16.14 | Octacosane | Straight chain alkane | 12408 | C28H58 |
| 44 | 16.28 | Sebacic acid | Alpha, omega-dicarboxylic acid | 5192 | C10H18O4 |
| 45 | 16.34 | Dodecane | Straight chain alkane | 8182 | C12H26 |
| 46 | 16.43 | Tritriacontane | Straight chain alkane | 12411 | C33H68 |
| 47 | 16.43 | Phytol | Terpenoids | 5366244 | C20H40O |
| 48 | 18.09 | Myristic Acid | Saturated fatty acid | 11005 | C14H28O2 |
| 49 | 18.52 | Oleic acid | Fatty acid | 445639 | C18H34O2 |
| 50 | 18.63 | Stearyl alcohol | Fatty alcohol | 8221 | C18H38O |
| 51 | 20.27 | Nonacosane | Straight chain alkane | 12409 | C29H60 |
| 52 | 21.37 | cis-Vaccenic acid | Fatty acid | 5282761 | C18H34O2 |
| 53 | 21.79 | Palmitic acid | Saturated fatty acid | 985 | C16H32O2 |
| 54 | 22.87 | cis-Farnesol | Terpenoids | 1549107 | C15H26O |
| 55 | 24.97 | Margaric acid | Saturated fatty acid | 10465 | C17H34O2 |
| 56 | 25.72 | Linoleic acid | Unsaturated fatty acid | 5280450 | C18H32O2 |
| 57 | 26.56 | Octadecadienoic acid | Unsaturated fatty acid | 3931 | C18H32O2 |

**Table 4S**: Acute toxicity study: Hematological parameters in rats treated with *Piper longum* ethanolic extract and hexane fraction.

| **S. No.** | **Treatment Group** | **Control** | **PLE**  **(2000mg/kg)** | **PLE-H**  **(2000mg/kg)** | **Control** | **PLE**  **(2000mg/kg)** | **PLE-H**  **(2000mg/kg)** |
| --- | --- | --- | --- | --- | --- | --- | --- |
|  |  | **Male** | | | **Female** | | |
| 1 | Haemoglobin (gm/dl) | 13.66±1.04 | 14.16±0.76 | 13.46±0.81 | 13.33±1.06 | 14.26±0.97 | 12.66±0.51 |
| 2 | Platelet Count (lacs/mm^3^) | 8.56±1.07 | 7.44±1.41 | 8.59±1.11 | 7.59±1.25 | 7.90±1.78 | 7.69±1.32 |
| 3 | RBC (10^6^/mm^3^) | 4.98±0.14 | 4.83±0.18 | 5.16±0.17 | 4.68±0.21 | 4.55±0.25 | 4.28±0.25 |
| 4 | PCV (%) | 42.93±3.07 | 43.2±2.74 | 40.23±1.28 | 40.0±1.32 | 40.39±1.79 | 39.13±2.0 |
| 5 | MCV (fL) | 86.07±5.15 | 89.32±2.71 | 77.97±1.2 | 85.55±5.46 | 88.97±8.76 | 91.41±2.56 |
| 6 | MCH (pg) | 27.38±1.32 | 29.37±2.68 | 26.12±1.88 | 28.56±3.33 | 31.29±0.83 | 29.66±2.59 |
| 7 | TLC (10^9^/L) | 5.56±0.5 | 5.03±0.41 | 6.16±0.6 | 7.33±0.45 | 8.46±0.47 | 8.0±0.72 |
|  | **Differential leukocyte count** | | | | | | |
| 8 | Neutrophil | 35.56±5.17 | 32.26±5.15 | 33.76±4.72 | 35.76±3.36 | 33.23±1.95 | 30.46±1.97 |
| 9 | Lymphocyte | 61.07±4.99 | 63.62±5.61 | 62.07±5.27 | 61.02±3.61 | 63.12±2.16 | 66.03±1.73 |
| 10 | Eosinophil | 1.4±0.12 | 1.41±0.17 | 1.64±0.16 | 1.31±0.1 | 1.39±0.13 | 1.29±0.13 |
| 11 | Monocyte | 1.7±0.36 | 2.36±0.35 | 2.13±0.35 | 1.5±0.3 | 1.8±0.26 | 1.73±0.2 |
| 12 | Basophil | 0.26±0.05 | 0.33±0.05 | 0.38±0.06 | 0.39±0.07 | 0.45±0.05 | 0.47±0.08 |

Values are expressed as mean ± SD (n = 5 per group). One-way ANOVA followed by Dunnett’s test. RBC= Red Blood Cell count, PCV= Packed Cell Volume, MCV=Mean Corpuscular Volume, MCH=Mean Corpuscular Hemoglobin, PLE: *Piper longum* ethanolic extract; PLE-H: Hexane fraction of *Piper longum.*

**Table 5S**: Acute toxicity study: Serum biochemical analysis in rats administered with *Piper longum* ethanolic extract and hexane fraction.

| **S. No.** | **Treatment Group** | **Control** | **PLE**  **(2000mg/kg)** | **PLE-H**  **(2000mg/kg)** | **Control** | **PLE**  **(2000mg/kg)** | **PLE-H**  **(2000mg/kg)** |
| --- | --- | --- | --- | --- | --- | --- | --- |
|  |  | **Male** | | | **Female** | | |
| 1 | ALP (U/I) | 102.71±6.63 | 103.34±6.98 | 105.95±8.36 | 95.35±6.96 | 92.33±7.16 | 95.78±5.18 |
| 2 | AST (U/L) | 111.12±4.14 | 112.41±4.34 | 110.16±4.92 | 114.83±2.18 | 111.39±2.51 | 112.22±5.71 |
| 3 | ALT (U/I) | 36.69±1.43 | 32.74±1.80 | 34.39±2.20 | 32.33±1.41 | 30.25±1.27 | 33.11±1.37 |
| 4 | Bilirubin Total (mg/dl) | 0.35±0.03 | 0.32±0.03 | 0.29±0.01 | 0.23±0.03 | 0.29±0.02 | 0.25±0.03 |
| 5 | Bilirubin Direct (mg/dl) | 0.073±0.007 | 0.077±0.004 | 0.066±0.007 | 0.077±0.006 | 0.073±0.005 | 0.082±0.006 |
| 6 | BUN (mg/dl) | 40.51±4.80 | 36.43±2.54 | 35.05±2.65 | 43.01±2.45 | 41.81±2.52 | 43.85±2.96 |
| 7 | Uric acid (mg/dl) | 1.89±0.12 | 1.81±0.15 | 2.08±0.20 | 1.92±0.09 | 2.04±0.08 | 1.75±0.17 |
| 8 | Creatinine (mg/dl) | 0.35±0.045 | 0.27±0.032 | 0.33±0.032 | 0.37±0.036 | 0.35±0.04 | 0.35±0.037 |
| 9 | Total Protein (g/dl) | 6.81±0.21 | 7.24±0.25 | 7.43±0.41 | 7.04±0.53 | 7.54±0.29 | 7.51±0.35 |
| 10 | Albumin (g/dl) | 3.30±0.22 | 3.27±0.27 | 3.46±0.31 | 3.12±0.27 | 3.05±0.41 | 3.25±0.34 |
| 11 | Calcium (mg/dl) | 9.81±0.35 | 9.53±0.20 | 9.81±0.25 | 9.10±0.35 | 9.51±0.40 | 9.04±0.35 |
| 12 | Glucose (mg/dl) | 106.36±6.90 | 97.24±9.86 | 110.98±12.92 | 109.7±9.41 | 106.44±9.56 | 103.9±7.29 |
| 13 | Total Cholesterol (mg/dl) | 61.55±4.05 | 63.14±3.28 | 60.29±3.77 | 57.94±3.07 | 56.09±4.00 | 60.55±5.77 |
| 14 | Triglyceride (mg/dl) | 30.51±2.92 | 33.11±2.23 | 32.23±1.99 | 35.54±2.74 | 32.91±1.90 | 35.73±2.35 |
| 15 | HDL-C (mg/dl) | 64.85±2.02 | 65.97±3.00 | 64.57±2.23 | 65.31±2.78 | 65.28±1.80 | 66.63±2.98 |
| 16 | VLDL (mg/dl) | 5.06±0.38 | 5.49±0.37 | 5.35±0.33 | 5.90±0.45 | 5.46±0.31 | 5.93±0.39 |

Values are expressed as mean ± SD (n = 5 per group). One-way ANOVA followed by Dunnett’s test. ALP= alkaline phosphatase, AST= aspartate aminotransferase, ALT=alanine aminotransferase, BUN= blood urea nitrogen, Creat=Creatinine, HDL=High Density Lipoprotein, VLDL= Very-Low-Density Lipoprotein, PLE: *Piper longum* ethanolic extract; PLE-H: Hexane fraction of *Piper longum.*

**Table 6S**: Sub-acute toxicity study: Hematological parameters in rats administered with *Piper longum* ethanolic extract and hexane fraction for 28 days.

| S. No. | Treatment | Haemoglobin (gm/dl) | Platelet Count (lacs/mm^3^) | RBC (10^6^/mm^3^) | PCV (%) | MCV (fL) | MCH (pg) |
| --- | --- | --- | --- | --- | --- | --- | --- |
| Male | | | | | | | |
| 1 | Control | 13.92±0.58 | 8.15±1.43 | 5.44±0.30 | 46.4±2.81 | 85.28±5.21 | 25.61±1.68 |
| 2 | PLE 200 mg/kg | 13.88±0.72 | 7.58±1.09 | 5.20±0.22 | 46.06±1.93 | 88.62±4.34 | 26.73±1.96 |
| 3 | PLE 400 mg/kg | 14.1±0.56 | 8.21±1.37 | 5.89±0.16* | 45.76±2.52 | 77.78±5.76 | 23.95±1.20 |
| 4 | PLE 800 mg/kg | 13.86±0.55 | 7.80±1.19 | 5.49±0.24 | 44.68±2.31 | 81.43±6.06 | 25.25±1.21 |
| 5 | PLE-H 200 mg/kg | 14.64±0.42 | 8.04±1.25 | 5.34±0.26 | 43.28±2.29 | 81.24±7.44 | 27.47±2.00 |
| 6 | PLE-H 400 mg/kg | 14.14±0.48 | 7.90±1.01 | 5.22±0.19 | 46.28±1.57 | 88.70±4.75 | 27.11±1.80* |
| 7 | PLE-H 800 mg/kg | 14±0.66 | 8.35±1.49 | 5.60±0.25 | 47.4±2.83 | 84.71±5.67 | 25.05±2.02 |
| Female | | | | | | | |
| 1 | Control | 12.46±0.63 | 8.18±1.39 | 5.24±0.24 | 38.26±1.54 | 72.99±4.11 | 23.81±1.92 |
| 2 | PLE 200 mg/kg | 13.4±1.39 | 7.25±1.16 | 5.28±0.21 | 37.2±2.03 | 70.50±5.10 | 25.34±2.28 |
| 3 | PLE 400 mg/kg | 12.76±1.00 | 7.88±1.27 | 5.32±0.15 | 38.64±1.55 | 72.90±3.12 | 24.09±2.24 |
| 4 | PLE 800 mg/kg | 11.82±083 | 7.88±1.17 | 4.80±0.16** | 38.64±1.60 | 80.47±4.49 | 24.59±1.35 |
| 5 | PLE-H 200 mg/kg | 12.92±0.85 | 8.37±1.35 | 4.93±0.18 | 37.80±1.35 | 76.72±3.18 | 26.25±2.19 |
| 6 | PLE-H 400 mg/kg | 13.35±0.52 | 8.17±1.00 | 4.92±0.18 | 38.26±1.71 | 76.89±4.80 | 27.11±1.27* |
| 7 | PLE-H 800 mg/kg | 12.16±0.50 | 8.14±1.31 | 5.47±0.18 | 40.10±2.32 | 73.45±6.30 | 22.24±0.95 |

Values are expressed as mean ± SD (n = 5 per group). One-way ANOVA followed by Dunnett’s test. Significant differences at *p < 0.05 and **p < 0.01 compared with the normal control group. RBC= Red Blood Cell count, PCV= Packed Cell Volume, MCV=Mean Corpuscular Volume, MCH=Mean Corpuscular Hemoglobin, PLE: *Piper longum* ethanolic extract; PLE-H: Hexane fraction of *Piper longum.*

**Table 7S**: Sub-acute toxicity study: Hematological parameters in rats administered with *Piper longum* ethanolic extract and hexane fraction for 28 days.

| S. No | Treatment Groups | TLC  (10^9^/L) | Differential Leucocytic Count (%) | | | | |
| --- | --- | --- | --- | --- | --- | --- | --- |
|  |  |  | Neutrophil | Lymphocyte | Eosinophil | Monocyte | Basophil |
| Male | | | | | | | |
| 1 | Control | 9.04±0.79 | 23.78±3.17 | 71.62±3.58 | 1.14±0.19 | 2.94±0.64 | 0.50±0.05 |
| 2 | PLE 200 mg/kg | 8.04±0.71 | 29.06±3.09 | 65.34±3.20* | 1.21±0.15 | 3.88±0.5* | 0.50±0.07 |
| 3 | PLE 400 mg/kg | 8.36±0.87 | 24.66±3.69 | 70.00±3.82 | 1.00±0.15 | 3.82±0.46 | 0.51±0.06 |
| 4 | PLE 800 mg/kg | 9.98±0.88 | 19.16±2.29 | 75.80±2.49 | 1.12±0.18 | 3.46±0.45 | 0.45±0.09 |
| 5 | PLE-H 200 mg/kg | 9.8±1.08 | 22.22±2.68 | 72.88±3.09 | 0.86±0.11* | 3.66±0.51 | 0.37±0.07* |
| 6 | PLE-H 400 mg/kg | 9.2±1.06 | 23.16±3.58 | 72.64±3.51 | 1.0±0.12 | 2.8±0.52 | 0.40±0.05 |
| 7 | PLE-H 800 mg/kg | 11.0±0.82** | 27.2±3.92 | 68.63±3.38 | 1.16±0.11 | 2.62±0.55 | 0.37±0.06* |
| Female | | | | | | | |
| 1 | Control | 8.16±1.06 | 25.46±3.87 | 70.42±3.79 | 1.22±0.07 | 2.4±0.48 | 0.31±0.06 |
| 2 | PLE 200 mg/kg | 8.04±0.72 | 29.96±1.88 | 65.61±1.52 | 1.29±0.08 | 2.82±0.41 | 0.31±0.04 |
| 3 | PLE 400 mg/kg | 7.74±1.21 | 23.04±2.71 | 72.11±2.86 | 1.27±0.05 | 3.22±0.54** | 0.34±0.04 |
| 4 | PLE 800 mg/kg | 7.28±0.84 | 22.32±3.64 | 72.92±4.18 | 1.31±0.11 | 3.12±0.58 | 0.32±0.06 |
| 5 | PLE-H 200 mg/kg | 7.22±0.63 | 23.32±2.47 | 72.01±2.42 | 1.18±0.18 | 3.14±0.49 | 0.34±0.06 |
| 6 | PLE-H 400 mg/kg | 7.58±0.87 | 25.24±3.05 | 70.67±3.17 | 1.05±0.16 | 2.74±0.43 | 0.29±0.04 |
| 7 | PLE-H 800 mg/kg | 9.26±0.93 | 32.44±3.38** | 63.24±3.53** | 1.16±0.11 | 2.92±0.4 | 0.23±0.04 |

Values are expressed as mean ± SD (n = 5 per group). One-way ANOVA followed by Dunnett’s test. Significant differences at *p < 0.05 and **p< 0.01 compared with the normal control group, respectively. TLC= Total leukocyte count, PLE: *Piper longum* ethanolic extract; PLE-H: Hexane fraction of *Piper longum.*

**Table 8S**: Recovery/ Withdrawal study: Hematological parameters in rats treated with *Piper longum* ethanolic extract and hexane fraction for 28 days.

| **S. No.** | **Treatment Group** | **Control** | **PLE**  **800 mg/kg** | **PLE-H**  **800 mg/kg** | **Control** | **PLE**  **800 mg/kg** | **PLE-H**  **800 mg/kg** |
| --- | --- | --- | --- | --- | --- | --- | --- |
|  |  | **Male** | | | **Female** | | |
| 1 | Haemoglobin (gm/dl) | 14.9±0.73 | 14.94±0.62 | 15.78±0.56 | 14.22±0.73 | 13.26±0.62 | 13.24±0.89 |
| 2 | Platelet Count (lacs/mm^3^) | 7.94±1.07 | 7.73±1.04 | 7.73±1.02 | 8.00±1.47 | 7.93±1.29 | 7.32±1.08 |
| 3 | RBC (10^6^/mm^3^) | 5.38±0.19 | 5.22±0.21 | 5.21±0.18 | 4.72±0.19 | 5.04±0.17* | 5.04±0.22* |
| 4 | PCV (%) | 43.08±2.51 | 39.92±0.21* | 43.42±2.01 | 41.80±1.37 | 41.66±2.07 | 42.44±1.76 |
| 5 | MCV (fL) | 80.11±4.73 | 76.46±2.98 | 83.43±5.08 | 88.65±4.84 | 82.54±2.50 | 84.20±4.26 |
| 6 | MCH (pg) | 27.70±1.35 | 28.59±0.87 | 30.31±1.52* | 30.18±2.52 | 26.27±0.45* | 26.32±2.71* |
| 7 | TLC (10^9^/L) | 6.22±0.79 | 5.42±0.46 | 5.84±0.55 | 7.14±0.67 | 6.92±0.66 | 8.24±0.53* |
| 8 | Neutrophil | 25.96±3.87 | 30.74±3.13 | 31.32±3.25 | 27.74±2.52 | 24.4±3.08 | 27.72±2.30 |
| 9 | Lymphocyte | 70.56±3.95 | 65.28±3.52 | 64.89±3.02* | 68.93±2.31 | 71.88±2.84 | 68.90±2.23 |
| 10 | Eosinophil | 1.02±0.19 | 1.02±0.14 | 1.08±0.13 | 1.20±0.83 | 1.27±0.08 | 1.08±0.05* |
| 11 | Monocyte | 2.12±0.25 | 2.62±0.34 | 2.46±0.38 | 1.8±0.36 | 2.12±0.42 | 2.02±0.32 |
| 12 | Basophil | 0.34±0.08 | 0.33±0.06 | 0.25±0.03 | 0.31±0.05 | 0.32±0.06 | 0.27±0.04 |

Values are expressed as mean ± SD (n = 5 per group). One-way ANOVA followed by Dunnett’s test. Significant differences at *p < 0.05 compared with the normal control group. RBC= Red Blood Cell count, PCV= Packed Cell Volume, MCV=Mean Corpuscular Volume, MCH=Mean Corpuscular Hemoglobin, TLC= Total leukocyte count, PLE: *Piper longum* ethanolic extract; PLE-H: Hexane fraction of *Piper longum.*

**Table 9S**: Recovery/Withdrawal study: Serum biochemical analysis in rats administered with *Piper longum* ethanolic extract and hexane fraction.

| **S. No.** | **Treatment Group** | **Control** | **PLE**  **800 mg/kg** | **PLE-H**  **800 mg/kg** | **Control** | **PLE**  **800 mg/kg** | **PLE-H**  **800 mg/kg** |
| --- | --- | --- | --- | --- | --- | --- | --- |
|  |  | **Male** | | | **Female** | | |
| 1 | ALP (U/I) | 128.81±10.30 | 120.98±7.12 | 119.46±9.11 | 122.36±7.58 | 109.18±6.44 | 109.69±7.12 |
| 2 | AST (U/L) | 113.63±4.53 | 111.13±3.11 | 120.17±3.01* | 112.70±5.11 | 114.26±2.56 | 119.86±4.93* |
| 3 | ALT (U/I) | 38.10±1.94 | 40.28±1.58 | 42.68±1.62** | 32.13±1.88 | 33.14±1.91 | 37.14±2.51** |
| 4 | Bilirubin Total (mg/dl) | 0.45±0.036 | 0.38±0.035* | 0.38±0.05* | 0.25±0.02 | 0.26±0.034 | 0.30±0.028* |
| 5 | Bilirubin Direct (mg/dl) | 0.064±0.004 | 0.071±0.007 | 0.071±0.005 | 0.091±0.007 | 0.087±0.006 | 0.091±0.006 |
| 6 | BUN (mg/dl) | 41.57±4.71 | 42.83±1.42 | 45.50±3.20 | 45.26±3.10 | 43.22±2.46 | 45.46±2.34 |
| 7 | Uric acid (mg/dl) | 2.17±0.16 | 2.20±0.13 | 2.14±0.24 | 1.99±0.17 | 1.76±0.29 | 1.99±0.14 |
| 8 | Creatinine (mg/dl) | 0.45±0.04 | 0.49±0.05 | 0.44±0.04 | 0.42±0.046 | 0.44±0.034 | 0.45±0.043 |
| 9 | Total Cholesterol (mg/dl) | 64.01±3.41 | 66.25±3.44 | 66.88±4.12 | 63.95±5.12 | 69.65±3.84 | 68.38±4.10 |
| 10 | Triglyceride (mg/dl) | 36.26±2.85 | 33.66±1.75 | 34.66±2.45 | 37.81±1.96 | 36.96±2.26 | 35.34±1.55 |
| 11 | HDL-C (mg/dl) | 63.98±2.68 | 65.93±2.66 | 64.21±3.00 | 66.97±2.02 | 64.93±2.80 | 65.96±3.66 |
| 12 | VLDL (mg/dl) | 6.01±0.47 | 5.58±0.29 | 5.75±0.40 | 6.27±0.32 | 6.13±0.37 | 5.86±0.25 |
| 13 | Total Protein (g/dl) | 7.03±0.29 | 7.18±0.27 | 7.40±0.33 | 6.99±0.39 | 7.25±0.48 | 6.94±0.31 |
| 14 | Albumin (g/dl) | 3.87±0.19 | 3.57±0.26 | 3.49±0.29 | 3.08±0.21 | 3.42±0.24 | 3.25±0.21 |
| 15 | Calcium (mg/dl) | 9.10±0.47 | 9.64±0.36 | 9.32±0.50 | 9.28±0.32 | 9.53±0.42 | 9.34±0.45 |
| 16 | Glucose (mg/dl) | 107.29±8.84 | 110.60±9.92 | 113.38±9.01 | 112.56±8.81 | 110.37±9.20 | 114.99±10.42 |

Values are expressed as mean ± SD (n = 5 per group). One-way ANOVA followed by Dunnett’s test. ALP= alkaline phosphatase, AST= aspartate aminotransferase, ALT=alanine aminotransferase, BUN= blood urea nitrogen, Creat=Creatinine, HDL=High Density Lipoprotein, VLDL= Very-Low-Density Lipoprotein, PLE: *Piper longum* ethanolic extract; PLE-H: Hexane fraction of *Piper longum.* Significant differences at *p< 0.05 and **p< 0.01 compared with the normal control group.

**Table 10S**: Histological criteria, severity and score to assess histopathological lesion of liver tissue

| S. No. | Histological criteria | Severity and Score |
| --- | --- | --- |
| 1 | Steatosis | Absent= 0  Mild=1  Moderate=2  Severe=3  Intensive=4 |
| 2 | Hyperplasia of bile duct |  |
| 3 | Necrosis/Apoptosis |  |
| 4 | Sinusoidal dilatation |  |
| 5 | Inflammatory cells |  |
| 6 | Congestion in the portal vein |  |
| 7 | Dilatation of the central vein |  |

**Figure Legends**

**Supplementary Figure 1S**: Quantification of Piperine and Piperlongumine by HPTLC: A. Overlay spectra of Piperine and Piperlongumine; B. Chromatograms; C: Peak of standard Piperine and Piperlongumine; D: Peaks of Piperine and Piperlongumine in Chemotype of *Piper longum* ethanolic extract; E: Peaks of Piperine and Piperlongumine in Chemotype of *Piper longum* hexane fraction

**Supplementary Figure 2S**: Qualitative analysis of phytoconstituents in ethanol extract of *Piper longum* by UPLC-MS

**Supplementary Figure 3S**: Phytochemical analysis and identification of major compounds in hexane fraction of *Piper longum* by GCMS
